# Feasibility and acceptability of an electronic immunization registry in urban Bangladesh

**DOI:** 10.1371/journal.pone.0352972

**Published:** 2026-07-02

**Authors:** Md. Jawadul Haque, A. K. M. Kamruzzaman, Rajendra Bohara, Chiranjit Das, Md. Tarjumanul Haque Sanij, Abul Fazal Md. Shahabuddin Khan, F. A. M. Anjuman Ara Begum, Nazim Uzzaman

**Affiliations:** 1 Rajshahi Medical University, Rajshahi, Bangladesh; 2 World Health Organization, Rajshahi, Bangladesh; 3 Rajshahi Medical College Hospital, Rajshahi, Bangladesh; 4 Directorate General of Health Services, Rajshahi, Bangladesh; 5 Rajshahi City Corporation, Rajshahi, Bangladesh; Kintampo Health Research Centre, GHANA

## Abstract

**Introduction:**

Electronic Immunization Registries (EIRs) are increasingly being implemented in low- and middle-income countries (LMICs) to support immunization tracking and data management. In Bangladesh, an EIR was introduced in Rajshahi City Corporation (RCC) to strengthen routine immunization services. However, evidence on user perceptions and operational implementation in this context remains limited. We assessed perceptions related to the feasibility and acceptability of the EIR among caregivers and healthcare providers (HCPs) in an urban pilot setting.

**Methods:**

We conducted a cross-sectional study between July and September 2024 in RCC, Bangladesh. Data were collected from caregivers of children receiving vaccinations and HCPs involved in immunization service delivery using a pretested semi-structured questionnaire. Perceptions of EIR-supported services were assessed using a 5-point Likert scale. Responses to open-ended questions were reviewed and summarized descriptively. This study was designed as a descriptive assessment and was not intended to evaluate the effectiveness or causal impact of the EIR system.

**Results:**

A total of 321 participants were included, comprising 305 caregivers and 16 HCPs. Electronic registration was conducted primarily at the Expanded Program on Immunization centers (87%), with additional outreach through house-to-house visits. Reported vaccination uptake among children registered in the EIR system was high within the study sample. Caregivers and HCPs reported generally positive perceptions of EIR-supported services. Most respondents selected ‘satisfied’ or ‘highly satisfied’ responses across assessed items. HCPs also reported positive experiences, particularly in relation to record management and data accessibility, while noting operational challenges such as intermittent internet connectivity and device-related limitations.

**Conclusions:**

The EIR was perceived as acceptable by caregivers and HCPs in this urban pilot setting. Further studies are needed to assess effectiveness, implementation challenges, and scalability across diverse settings.

## Introduction

Immunization remains one of the most successful and cost-effective public health interventions globally, preventing an estimated 4.4 million deaths each year [1,2]. Despite these gains, coverage gaps persist, particularly in low- and middle-income countries (LMICs), where health system fragmentation, inadequate monitoring, and delayed reporting hinder timely vaccination [3,4]. The World Health Organization’s Immunization Agenda 2030 (IA2030) prioritizes strengthening data systems and digital health innovations to achieve equitable, timely, and complete coverage [5].

Bangladesh has made substantial progress in immunization since the launch of its Expanded Program on Immunization (EPI) in 1979, with coverage of key childhood vaccines reaching around 90% and marked reductions in vaccine-preventable diseases [6]. However, challenges remain in urban and peri-urban settings where rapid population growth, informal settlements, and migration complicate outreach and data tracking [7]. Coverage Evaluation Surveys 2023 revealed gaps between crude and valid vaccination coverage in Bangladesh, where children may receive vaccines but not according to the recommended schedule [8]. These gaps may be related to delayed vaccination, missed doses, and limitations in record-keeping and tracking systems.

Electronic Immunization Registries (EIRs) may support immunization programs by improving individual-level tracking, strengthening data availability, and enabling reminder systems [9–11]. Global experiences illustrate the benefits of transitioning from paper-based to digital systems: Vietnam’s National Immunization Information System has streamlined vaccine workflows and integrated SMS-based reminders [12]; the ‘Better Immunization Data’ initiative in Tanzania and Zambia demonstrated improved timeliness and data quality [13]; and Pakistan’s ‘Zindagi Mehfooz’ registry showed significant improvements in immunization tracking and engagement [14]. Similarly, evidence from Kenya, Brazil, and other LMICs highlights how digital registries enable targeted outreach, reduce dropout, and enhance planning [15].

In Bangladesh, WHO supported the introduction of an EIR in Rajshahi City Corporation (RCC) in 2019 to address gaps in immunization coverage and tracking [16]. This pilot system introduced unique identifiers, real-time reporting, and SMS reminders to enhance service delivery and improve data-driven decision-making [16]. Although EIRs have been introduced in selected settings in Bangladesh, available evidence is largely limited to programmatic descriptions of implementation. Empirical evidence on user-level perceptions, including feasibility and acceptability among caregivers and HCPs, remains limited. Understanding these aspects is important to inform how such systems function in routine service delivery. This study explored user perceptions and operational experiences related to the feasibility and acceptability of the EIR among caregivers and HCPs in RCC.

## Materials and Methods

### Study design and setting

A cross-sectional survey was conducted from July to September 2024 in RCC, Bangladesh. This study was designed as a descriptive assessment of perceptions related to the feasibility and acceptability of the EIR system among caregivers and HCPs. The study was not designed to evaluate effectiveness or causal impact of the EIR system. The assessment reflects user perceptions and reported operational experiences during the 2024 data collection period rather than the full implementation period since the EIR was introduced in 2019. The EIR system included unique digital identifiers for each child, real-time data entry using tablet devices, and automated SMS reminders to caregivers. Caregivers were recipients of EIR-supported services, while HCPs were the direct users of the digital platform.

### Study participants

Adult caregivers residing in different wards of RCC who had children receiving routine immunization services, and HCPs involved in immunization service delivery, were eligible for inclusion in the study. The minimum required sample size for caregivers was initially estimated using the single-population proportion formula. In the absence of prior data on the feasibility or acceptability of EIRs in Bangladesh, the prevalence of caregivers lacking vaccination records (16%) [17] was used as an operationally relevant proxy indicator of baseline registration completeness. An initial estimate of approximately 207 caregivers was used to guide recruitment. Given the descriptive and operational nature of the study and the need to include participants with direct experience of the RCC EIR, purposive sampling was used to recruit caregivers attending EPI sessions. HCPs involved in EIR-supported immunization service delivery at the study site were approached for participation. As participants had already been exposed to EIR-supported services, the findings may reflect the perceptions of relatively engaged users and may overestimate satisfaction and perceived benefits.

### Data collection

A semi-structured questionnaire containing both closed- and open-ended questions was used to collect information on demographics, registration timelines, satisfaction levels, and perceived benefits and challenges of using the EIR. The questionnaire was pretested among a small group of caregivers and HCPs to assess clarity, comprehension, and flow. Minor modifications were made prior to data collection. The questionnaire included sections tailored to caregivers and HCPs; caregiver items focused on experiences of EIR-supported services, while HCP items addressed system use, data entry processes, and operational challenges. A 5-point Likert scale was used to assess the perceptions related to EIR-supported services, with response options of not at all satisfied (1), dissatisfied (2), neutral (3), satisfied (4), and highly satisfied (5). These measures were used to assess perceptions related to the feasibility and acceptability of EIR-supported services, with satisfaction interpreted as one component of acceptability.

### Data analysis

Quantitative data were analyzed using Statistical Package for the Social Sciences (SPSS), version 24.0 (IBM Corp., Armonk, NY, USA). Descriptive statistics, including frequencies and percentages, were used to summarize participant characteristics, registration patterns, vaccination status, satisfaction responses, and reported challenges. The analysis was descriptive and aligned with the exploratory nature and objectives of the study. Responses on the Likert-scale items were analyzed individually and were not combined into a composite score. Responses to open-ended questions were reviewed and descriptively summarized to identify commonly reported issues and experiences.

### Ethics

Ethical approval was obtained from the Institutional Review Board of Islami Bank Medical College (Ref: IBMC,R/IRB/2024/04/01). Written informed consent was obtained from all participants. All data were anonymized prior to analysis, and no personally identifiable information was included in the dataset used for this study.

## Results

### Demographics

A total of 321 participants were included in the study, comprising 305 caregivers and 16 HCPs. Among caregivers, most respondents were mothers (78%), followed by fathers (21%) and grandparents (1%). Among HCPs, vaccinators represented 56%, followed by vaccination supervisors (25%) and community health workers (19%).

### Electronic registration and vaccination coverage

Electronic registration was conducted primarily at EPI centers (87%), with the remaining carried out during house-to-house visits, indicating that facility-based registration was the dominant approach, supported by outreach services. Reported vaccination uptake among children registered in the EIR system was high within the study sample (Table 1). These estimates reflect reported vaccination status among children registered in the EIR system and should not be interpreted as population-level vaccination coverage.

**Table 1:** Electronic immunization registration and vaccination coverage.

Indicator	Number	Target achieved^a^
Electronic registration	305	100%
Registered within 45 days	268	88%
BCG vaccination	305	100%
Pentavalent 1–3	305	100%
MR‑1 vaccination	305	100%
MR‑2 vaccination	296	97%

^a^Percentages are calculated using the total number of respondents as the denominator.

### Caregiver and HCP satisfaction

Caregiver responses reflected perceptions of EIR-supported service delivery rather than direct interaction with the digital registry system. Among caregivers, most respondents reported ‘satisfied’ or ‘highly satisfied’ responses across all assessed items, including ease of accessing EIR-supported services, usefulness of SMS reminders, preference for EIR over manual registration, and the perceived benefits of EIR (Table 2). The proportion reporting ‘satisfied’ responses ranged from 59% to 78%, while ‘highly satisfied’ responses ranged from 16% to 22% across items. No respondents selected the ‘dissatisfied’ or ‘not at all satisfied’ response categories.

**Table 2:** Caregiver perceptions of EIR-supported services.

Statements	Highly Satisfied n (%)^a^	Satisfied n (%)	Neutral n (%)	Dissatisfied n (%)	Not at all satisfied n (%)
Ease of accessing EIR^b^-supported services	52 (17)	225 (74)	28 (9)	0 (0)	0 (0)
SMS reminders supporting timely child vaccination	50 (16)	191 (63)	64 (21)	0 (0)	0 (0)
Preference for using EIR over manual registration	52 (17)	181 (59)	72 (24)	0 (0)	0 (0)
Contribution of EIR to the EPI program	51 (17)	211 (69)	43 (14)	0 (0)	0 (0)
Provision of electronic vaccination certificates	66 (22)	239 (78)	0 (0)	0 (0)	0 (0)
Suitability of EIR for nationwide implementation	57 (19)	194 (63)	54 (18)	0 (0)	0 (0)

a Percentages are calculated using the total number of respondents as the denominator. Satisfaction was assessed using a 5-point Likert scale: 1 = not at all satisfied, 2 = dissatisfied, 3 = neutral, 4 = satisfied, and 5 = highly satisfied.

b EIR = electronic immunization registry.

Among HCPs, responses also indicated generally positive perceptions across all assessed items, including the ease of handling the software, the simplicity of learning and managing the process, preference for EIR over manual registration, and the ease of accessing immunization records (Table 3). Most HCPs reported ‘highly satisfied’ responses, with smaller proportions selecting ‘satisfied’ or ‘neutral’. The proportion reporting ‘highly satisfied’ responses ranged from 75% to 88%. One response indicated ‘dissatisfied’ for the item related to ease of learning and managing the system, and no respondents selected the ‘not at all satisfied’ category. Given the small number of HCPs (n = 16), these findings should be interpreted as indicative of provider perceptions within this setting.

**Table 3:** Healthcare provider perceptions of EIR-supported services.

Statements	Highly Satisfied n (%)^a^	Satisfied n (%)	Neutral n (%)	Dissatisfied n (%)	Not at all satisfied n (%)
Ease of handling EIR^b^ software	12 (75)	4 (25)	0 (0)	0 (0)	0 (0)
EIR process is easy to learn and manage	13 (81)	1 (6)	1 (6)	1 (7)	0 (0)
Preference for EIR over manual registration	14 (88)	1 (6)	1 (6)	0 (0)	0 (0)
Ease of accessing immunization records using EIR	14 (87)	2 (13)	0 (0)	0 (0)	0 (0)
EIR is beneficial for immunization service delivery	12 (75)	4 (25)	0 (0)	0 (0)	0 (0)

a Percentages are calculated using the total number of respondents as the denominator. Satisfaction was assessed using a 5-point Likert scale: 1 = not at all satisfied, 2 = dissatisfied, 3 = neutral, 4 = satisfied, and 5 = highly satisfied.

b EIR = electronic immunization registry

### Perceived challenges and benefits

HCPs reported several operational and technical challenges, most commonly slow internet connectivity and device-related limitations. Despite these challenges, providers highlighted advantages such as easier access to immunization records, reduced reliance on paper-based record keeping, and improved timeliness of report sharing.

## Discussion

### Summary of the findings

Our study provides a descriptive assessment of user perceptions and reported operational experiences related to an EIR in an urban pilot setting in Bangladesh. The system was implemented within routine immunization service delivery, and both caregivers and HCPs reported generally positive perceptions and experiences. Caregivers described EIR-supported services as convenient, particularly in relation to receiving vaccination-related information and reminders, while HCPs highlighted advantages in record management and data accessibility. However, operational challenges, including intermittent internet connectivity and device-related issues, were also noted. These findings should be interpreted primarily as descriptive perceptions from an urban pilot implementation context rather than as evidence of effectiveness or system-level impact. Caregivers were not direct users of the EIR system, but recipients of services supported by it. Their responses therefore reflect perceptions of EIR-supported immunization services, such as reminders, certificates, and access to vaccination information, rather than direct interaction with the digital registry itself.

### Strengths and limitations

This study provides early evidence on user perceptions and operational experiences related to EIR-supported immunization services in Bangladesh. By combining structured survey findings with descriptive summaries of open-ended responses, the study provides insights into usability, perceived benefits, and operational challenges associated with EIR-supported services. The inclusion of caregivers and HCPs allowed assessment of perspectives from service recipients and direct system users. The study also contributes context-specific insights on user perceptions and operational experiences of EIR-supported immunization services in Bangladesh, which may help inform future implementation and evaluation efforts.

This study has several limitations. First, the cross-sectional design provides a snapshot of user perceptions and does not allow assessment of sustainability, dropout reduction, equity impacts, or changes over time. Second, the absence of a comparison group means that observed outcomes such as reported vaccination coverage and registration timeliness cannot be attributed solely to the EIR system. Third, participants were purposively selected and had already been exposed to EIR-supported services, which may have overestimated satisfaction and perceived benefits. Fourth, satisfaction measures relied on self-report and may be affected by recall or social desirability bias. Fifth, the number of HCPs was small (n = 16), restricting the range of provider responses captured. Finally, the study was conducted in a single urban pilot setting, and findings may not be transferable to rural or resource-constrained areas where infrastructure, staffing, and connectivity differ substantially. Caregiver findings should also be interpreted as perceptions of service delivery influenced by the EIR rather than direct evaluation of the digital registry system. Future studies should include longitudinal or comparative designs, objective registry audits, richer qualitative exploration of HCP experiences, and assessment across diverse geographic settings.

### Interpretation in the light of published papers

The reported registration timeliness of 88% within 45 days is consistent with findings from several LMIC settings implementing EIR systems. Similar findings have been reported in Tanzania, Zambia, and Vietnam, where EIRs were associated with early registration and follow-up [18]. SMS reminders, adopted in multiple LMIC settings, further strengthen adherence to vaccination schedules by reducing missed appointments and improving completion rates [19–21]. The generally positive perceptions reported in this study are consistent with evidence from Kenya and Pakistan, where registries were associated with better user engagement and perceived service quality [14,22].

Despite these achievements, several challenges persist. Technological barriers, including intermittent internet access, and device limitations, were reported by providers. These findings are broadly consistent with reports from Zambia and Pakistan, where inadequate infrastructure occasionally limited EIR functionality [14,23]. Addressing these challenges will require sustained investment in digital infrastructure, offline-capable systems, and refresher training for frontline staff. These issues are particularly important if EIR implementation is extended beyond urban settings to rural or resource-constrained areas. Evidence from other LMIC settings suggests that EIRs may support improved data use, service delivery, and immunization program planning [23–24]. However, implementation challenges related to infrastructure, workforce capacity, and system integration remain important considerations. Experiences from other settings, including Vietnam, highlight the importance of phased implementation and coordination across stakeholders when scaling EIR systems [25].

As Bangladesh continues efforts to strengthen equitable and timely immunization coverage, EIRs may support strengthening service delivery and monitoring. However, nationwide implementation will require coordinated investment in improving internet connectivity and speed, capacity building, and integration with broader health information systems. Leveraging EIR data for microplanning, outreach, and resource allocation could further reduce missed opportunities for vaccination and improve health outcomes.

### Conclusion

The EIR was perceived as acceptable by caregivers and HCPs in this urban pilot setting. However, the findings should be interpreted as descriptive evidence on user perceptions and operational experiences rather than evidence of effectiveness or scalability. Further studies are needed to assess implementation challenges, effectiveness, and scalability across diverse settings.

## Supporting information

S1 Dataset De-identified quantitative dataset underlying the findings of this study (Excel format). (XLSX)
