# Supplementary material for: Feasibility and acceptability of an electronic immunization registry in urban Bangladesh
Source: PLoS One. 2026 Jul 2;21(7):e0352972. doi: 10.1371/journal.pone.0352972 (PMC13327150; doi:10.1371/journal.pone.0352972)
Supplement: Attachment — Submitted filename: PONE-D-25-67616_reviewer_comments.pdf [file pone.0352972.s005.pdf]

PLOS One

Feasibility and acceptability of an electronic immunization registry in urban Bangladesh

--Manuscript Draft--

**Manuscript Number:**	PONE-D-25-67616
**Article Type:**	Research Article
**Full Title:**	Feasibility and acceptability of an electronic immunization registry in urban Bangladesh
**Short Title:**	Feasibility of an electronic immunization registry in Bangladesh
**Corresponding Author:**	Nazim Uzzaman, MBBS, PhD<br>Rajshahi Medical University<br>Rajshahi, BANGLADESH
**Keywords:**	Electronic immunization registry; vaccination coverage; childhood immunization; Bangladesh
**Abstract:**	Introduction<br><br>Electronic Immunization Registries (EIRs) are increasingly adopted in low- and middle-income countries (LMICs) to enhance vaccination data quality, improve tracking, and strengthen caregiver engagement. In 2019, an EIR was piloted in Rajshahi City Corporation (RCC), Bangladesh, to improve immunization monitoring and coverage. We assessed the feasibility of the EIR to provide evidence and inform policymakers on strategies for scaling up digital immunization systems in Bangladesh.<br><br>Methods<br><br>We conducted a cross-sectional study between July and September 2024 using a semi-structured questionnaire containing both closed- and open-ended questions. Quantitative data were analyzed using SPSS version 24.0, and qualitative responses were summarized descriptively.<br><br>Results<br><br>A total of 321 participants were included, comprising 305 caregivers and 16 healthcare providers (HCPs). Electronic registration was conducted mainly at the Expanded Program on Immunization centers (90%) and through house-to-house visits. Vaccination coverage was high, with 100% receiving BCG, Pentavalent 1–3, and MR-1 vaccines, while 97% received MR-2. Caregivers reported high satisfaction with the EIR, with 86% satisfied or highly satisfied and 83% supporting nationwide expansion. Similarly, 93% of HCPs expressed satisfaction, although slow internet and tablet malfunctions were noted as common challenges.<br><br>Conclusions<br><br>The RCC EIR improved registration timeliness, vaccination tracking, and caregiver satisfaction. Despite minor technological challenges, the system demonstrates feasibility, acceptability, and scalability for nationwide implementation.
**Order of Authors:**	Nazim Uzzaman, MBBS, PhD
	Md. Jawadul Haque, MBBS, PhD
	A K M Kamruzzaman
	Rajendra Bohara
	Chiranjit Das
	Md.Tarjumanul Haque
	Abul Fazal Md. Shahabuddin Khan
	F A M Anjuman Ara Begum

Powered by Editorial Manager® and ProduXion Manager® from Aries Systems Corporation

Additional Information:	
**Question**	**Response**
**Financial Disclosure**<br><br>Enter a financial disclosure statement that describes the sources of funding for the work included in this submission. Review the submission guidelines for detailed requirements. View published research articles from *PLOS ONE* for specific examples.<br><br>This statement is required for submission and **will appear in the published article** if the submission is accepted. Please make sure it is accurate.<br><br>**Funded studies**<br>Enter a statement with the following details:<br>• Initials of the authors who received each award<br>• Grant numbers awarded to each author<br>• The full name of each funder<br>• URL of each funder website<br>• Did the sponsors or funders play any role in the study design, data collection and analysis, decision to publish, or preparation of the manuscript?<br><br>Did you receive funding for this work?	The author(s) received no specific funding for this work.
**Competing Interests**<br><br>Use the instructions below to enter a competing interest statement for this submission. On behalf of all authors, disclose any competing interests that could be perceived to bias this work—acknowledging all financial support and any other relevant financial or non-financial competing interests.<br><br>This statement is required for submission and **will appear in the published article** if the submission is accepted. Please make sure it is accurate and that any funding sources listed in your Funding Information	The authors have declared that no competing interests exist.

later in the submission form are also declared in your Financial Disclosure statement.<br><br>View published research articles from *PLOS ONE* for specific examples.<br><br>**NO authors have competing interests**<br><br>Enter: *The authors have declared that no competing interests exist.*<br><br>**Authors with competing interests**<br><br>Enter competing interest details beginning with this statement:<br><br>*I have read the journal's policy and the authors of this manuscript have the following competing interests: [insert competing interests here]*<br><br>* typeset	
**Ethics Statement**<br><br>Enter an ethics statement for this submission. This statement is required if the study involved:<br><br>• Human participants<br>• Human specimens or tissue<br>• Vertebrate animals or cephalopods<br>• Vertebrate embryos or tissues<br>• Field research<br><br>Write "N/A" if the submission does not require an ethics statement.<br><br>General guidance is provided below. Consult the submission guidelines for detailed instructions. **Make sure that all information entered here is included in the Methods section of the manuscript.**	Ethical approval was obtained from the Ethics Review Board of Islami Bank Medical College, Rajshahi, Bangladesh, and written informed consent was obtained from all participants.

**Format for specific study types**<br><br>**Human Subject Research (involving human participants and/or tissue)**<br>• Give the name of the institutional review board or ethics committee that approved the study<br>• Include the approval number and/or a statement indicating approval of this research<br>• Indicate the form of consent obtained (written/oral) or the reason that consent was not obtained (e.g. the data were analyzed anonymously)<br><br>**Animal Research (involving vertebrate animals, embryos or tissues)**<br>• Provide the name of the Institutional Animal Care and Use Committee (IACUC) or other relevant ethics board that reviewed the study protocol, and indicate whether they approved this research or granted a formal waiver of ethical approval<br>• Include an approval number if one was obtained<br>• If the study involved *non-human primates*, add *additional details* about animal welfare and steps taken to ameliorate suffering<br>• If anesthesia, euthanasia, or any kind of animal sacrifice is part of the study, include briefly which substances and/or methods were applied<br><br>**Field Research**<br><br>Include the following details if this study involves the collection of plant, animal, or other materials from a natural setting:<br>• Field permit number<br>• Name of the institution or relevant body that granted permission	
**Data Availability**<br><br>Authors are required to make all data underlying the findings described fully available, without restriction, and from the time of publication. PLOS allows rare exceptions to address legal and ethical concerns. See the PLOS Data Policy and FAQ for detailed information.	Yes - all data are fully available without restriction

A Data Availability Statement describing where the data can be found is required at submission. Your answers to this question constitute the Data Availability Statement and **will be published in the article**, if accepted.<br><br>**Important:** Stating 'data available on request from the author' is not sufficient. If your data are only available upon request, select 'No' for the first question and explain your exceptional situation in the text box.<br><br>Do the authors confirm that all data underlying the findings described in their manuscript are fully available without restriction?	
**Describe where the data may be found in full sentences. If you are copying our sample text, replace any instances of XXX with the appropriate details.**<br><br>• If the data are **held or will be held in a public repository**, include URLs, accession numbers or DOIs. If this information will only be available after acceptance, indicate this by ticking the box below. For example: *All XXX files are available from the XXX database (accession number(s) XXX, XXX.).*<br>• If the data are all contained **within the manuscript and/or Supporting Information files**, enter the following: *All relevant data are within the manuscript and its Supporting Information files.*<br>• If neither of these applies but you are able to provide **details of access elsewhere**, with or without limitations, please do so. For example:<br><br>*Data cannot be shared publicly because of [XXX]. Data are available from the XXX Institutional Data Access / Ethics Committee (contact via XXX) for researchers who meet the criteria for access to confidential data.*<br><br>*The data underlying the results presented in the study are available from (include the name of the third party*	All relevant data are within the manuscript.

and contact information or URL).<br>• This text is appropriate if the data are owned by a third party and authors do not have permission to share the data.<br><br>* typeset	
Additional data availability information:	

Manuscript Click here to access/download;Manuscript;Manuscript.docx

Feasibility and acceptability of an electronic immunization registry in urban Bangladesh

Md. Jawadul Haque[1], A K M Kamruzzaman[2], Rajendra Bohara[2], Chiranjit Das[2], Md. Tarjumanul Haque Sanij[3], Abul Fazal Md. Shahabuddin Khan[4], F A M Anjuman Ara Begum[5], Nazim Uzzaman[1]*

[1]Rajshahi Medical University, Bangladesh

[2]World Health Organization, Bangladesh

[3]Rajshahi Medical College Hospital, Rajshahi, Bangladesh

[4]Directorate General of Health Services, Bangladesh

[5]Rajshahi City Corporation, Bangladesh

*Corresponding author

Email: nazim0700@gmail.com (NU)

Abstract

Introduction

Electronic Immunization Registries (EIRs) are increasingly adopted in low- and middle-income countries (LMICs) to enhance vaccination data quality, improve tracking, and strengthen caregiver engagement. In 2019, an EIR was piloted in Rajshahi City Corporation (RCC), Bangladesh, to improve immunization monitoring and coverage. We assessed the feasibility of the EIR to provide evidence and inform policymakers on strategies for scaling up digital immunization systems in Bangladesh.

Methods

We conducted a cross-sectional study between July and September 2024 using a semi-structured questionnaire containing both closed- and open-ended questions. Quantitative data were analyzed using SPSS version 24.0, and qualitative responses were summarized descriptively.

Results

A total of 321 participants were included, comprising 305 caregivers and 16 healthcare providers (HCPs). Electronic registration was conducted mainly at the Expanded Program on Immunization centers (90%) and through house-to-house visits. Vaccination coverage was high, with 100% receiving BCG, Pentavalent 1–3, and MR-1 vaccines, while 97% received MR-2. Caregivers reported high satisfaction with the EIR, with 86% satisfied or highly satisfied and 83% supporting nationwide expansion. Similarly, 93% of HCPs expressed satisfaction, although slow internet and tablet malfunctions were noted as common challenges.

Conclusions

The RCC EIR improved registration timeliness, vaccination tracking, and caregiver satisfaction. Despite minor technological challenges, the system demonstrates feasibility, acceptability, and scalability for nationwide implementation.

Keywords: Electronic immunization registry, vaccination coverage, childhood immunization, Bangladesh.

Introduction

Immunization remains one of the most successful and cost-effective public health interventions globally, preventing an estimated 4.4 million deaths each year [1, 2]. Despite these gains, coverage gaps persist, particularly in low- and middle-income countries (LMICs), where health system fragmentation, inadequate monitoring, and delayed reporting hinder timely vaccination [3, 4]. The World Health Organization's Immunization Agenda 2030 (IA2030) prioritizes strengthening data systems and digital health innovations to achieve equitable, timely, and complete coverage [5].

Bangladesh has made remarkable progress in immunization since the launch of its Expanded Program on Immunization (EPI) in 1979, achieving impressive reductions in vaccine-preventable diseases [6]. However, challenges remain in urban and peri-urban settings where rapid population growth, informal settlements, and migration complicate outreach and data tracking [7]. Coverage Evaluation Surveys 2023 revealed gaps between crude and valid vaccination coverage, underscoring the need for innovative solutions to improve monitoring, timeliness, and completion rates [8].

Electronic Immunization Registries (EIRs) have emerged as powerful tools in LMICs to improve data accuracy, enable individualized tracking, and facilitate automated reminders to caregivers [9-11]. Global experiences illustrate the benefits of transitioning from paper-based to digital systems: Vietnam's National Immunization Information System has streamlined vaccine workflows and integrated SMS-based reminders [12]; the 'Better Immunization Data' initiative in Tanzania and Zambia demonstrated improved timeliness and data quality [13]; and Pakistan's 'Zindagi Mehfooz' registry showed significant

improvements in immunization tracking and engagement [14]. Similarly, evidence from Kenya, Brazil, and other LMICs highlights how digital registries enable targeted outreach, reduce dropout, and enhance planning [15].

In Bangladesh, WHO supported the introduction of an EIR in Rajshahi City Corporation (RCC) in 2019 to address longstanding gaps in immunization coverage and tracking [16]. This pilot system introduced unique identifiers, real-time reporting, and SMS reminders to enhance service delivery and improve data-driven decision-making [16]. Despite growing interest in scaling up EIRs nationwide, empirical evidence on their outcomes in Bangladesh remains limited. This study evaluated the feasibility of the RCC EIR by assessing registration timeliness, vaccination coverage, caregiver and healthcare provider (HCP) satisfaction, and perceived barriers and enablers, providing evidence to inform digital health scale-up strategies and policy decisions aimed at achieving universal childhood immunization.

Materials and Methods

Study design and setting

A cross-sectional survey was conducted from July to September 2024 in RCC, Bangladesh.

Study participants

All adult caregivers residing in different wards of RCC and all HCPs involved in the vaccination program constituted the study population. The minimum required sample size for caregivers was estimated using the single-population proportion formula $n = Z^2pq/d^2$.

As no prior data were available on the feasibility or acceptability of EIRs in Bangladesh, we used the prevalence of caregivers lacking vaccination records (16%) [17] as a programmatically meaningful proxy indicator of baseline registration completeness. Using this estimate, with a 95% confidence level and a 5% margin of error, the calculated minimum sample size was approximately 207. Given the operational focus of the study and the need to include participants with direct exposure to the RCC EIR, purposive sampling was used to recruit information-rich caregivers and all HCPs involved in immunization service delivery.

Data collection

A semi-structured questionnaire containing both closed- and open-ended questions was used to collect information on demographics, registration timelines, satisfaction levels, and perceived benefits and challenges of using the EIR. A 5-point Likert scale was incorporated to assess respondents' satisfaction with the EIR, where 0 represented "not at all satisfied" and 5 represented "highly satisfied".

Data analysis

Quantitative data were analyzed using Statistical Package for the Social Sciences (SPSS) software, version 24.0 (IBM Corp., Armonk, NY, USA). Descriptive statistics, including frequencies, percentages, means and standard deviations, were computed to summarize the data. Qualitative data from the open-ended questions on perceived benefits and challenges were summarized and reported in a descriptive form.

Ethics

Ethical approval was obtained from the Ethics Review Board of Islami Bank Medical College, Rajshahi, Bangladesh, and written informed consent was obtained from all participants.

Results

Demographics

A total of 321 participants were included in the study, comprising 305 caregivers and 16 HCPs. Among caregivers, 78% were mothers, 21% fathers, and 1% grandparents. Among HCPs, vaccinators represented 56%, followed by vaccination supervisors (25%) and community health workers (19%).

Electronic registration and vaccination coverage

Electronic registration was conducted primarily at EPI centers (90%), with the remaining 10% carried out during house-to-house visits, indicating that facility-based registration was the dominant approach, supported by outreach services. All children in the study were electronically registered, and 88% were registered within 45 days of birth. Vaccination coverage was also high: 100% of children received BCG, Pentavalent 1–3, and MR-1 vaccines, while 97% received MR-2 (Table 1).

Table 1. Registration and vaccination coverage

Indicator	Number	Target achieved
Electronic registration	305	100%
Registered within 45 days	268	88%
BCG vaccination	305	100%
Pentavalent 1–3	305	100%
MR-1 vaccination	305	100%
MR-2 vaccination	296	97%

Caregiver and HCP satisfaction

Caregivers reported a high level of satisfaction with the EIR system. Overall, 86% were either highly satisfied or satisfied across all measured dimensions, including ease of use, the usefulness of SMS reminders, preference for EIR over manual registration, and the perceived benefits of EIR for the EPI. Furthermore, all caregivers expressed satisfaction with receiving a digital vaccination certificate, and 83% agreed that the system is suitable for nationwide implementation, with none reporting dissatisfaction. These findings highlight the strong acceptance of the EIR platform among city dwellers and its potential for broader scalability (see Table 2).

Table 2. Caregiver satisfaction with EIR system (5-point Likert scale)

Statements	Highly Satisfied n (%)	Satisfied n (%)	Neutral n (%)
Ease of using the EIR system	52 (17)	225 (74)	28 (9)
SMS reminders supporting timely child vaccination	50 (16)	191 (63)	64 (21)
Preference for using EIR over manual registration	52 (17)	181 (59)	72 (24)
Contribution of EIR to the EPI program	51 (17)	211 (69)	43 (14)
Provision of electronic vaccination certificates	66 (22)	239 (78)	0 (0)
Suitability of EIR for nationwide implementation	57 (19)	194 (63)	54 (18)

Similarly, HCPs also expressed high satisfaction with the EIR system. In total, 93% were either highly satisfied or satisfied across all assessed dimensions, including the ease of handling the software, the simplicity of learning and managing the process, the preference for EIR over manual registration, and the ease of accessing immunization records. Moreover, all HCPs agreed that the EIR system is beneficial for immunization service delivery. These findings demonstrate a strong endorsement of the EIR among HCPs and support its feasibility and scalability for broader implementation (see Table 3).

Table 3. HCP satisfaction with EIR system (5-point Likert scale)

Statements	Highly Satisfied n (%)	Satisfied n (%)	Neutral n (%)	Dissatisfied n (%)
Ease of handling EIR software	12 (75)	4 (25)	-	-
EIR process is easy to learn and manage	13 (81)	1 (6)	1 (6)	1 (7)
Preference for EIR over manual registration	14 (88)	1 (6)	1 (6)	-
Ease of accessing immunization records using EIR	14 (87)	2 (13)	-	-
EIR is beneficial for immunization service delivery	12 (75)	4 (25)	-	-

Perceived challenges and benefits

HCPs reported several technical and operational challenges during the EIR submission process, with the most common being slow internet connectivity (31%), and combined slow internet with tablet issues (37%) (Figure 3, Panel A). Tablet malfunctions were independently reported by 19%, while a smaller proportion mentioned that the process was time-consuming (6%) or involved duplication of work (6%).

Despite these challenges, HCPs highlighted multiple advantages of the EIR system (Figure 1, Panel B). The most frequently cited benefits included the ability to easily search vaccination records and track dropouts or missed doses (31%), the elimination of paper-based record-keeping (48%), and improved timely report sharing (25%). Overall, the findings demonstrated that although technical issues existed, HCPs recognized the value and efficiency of the EIR platform.

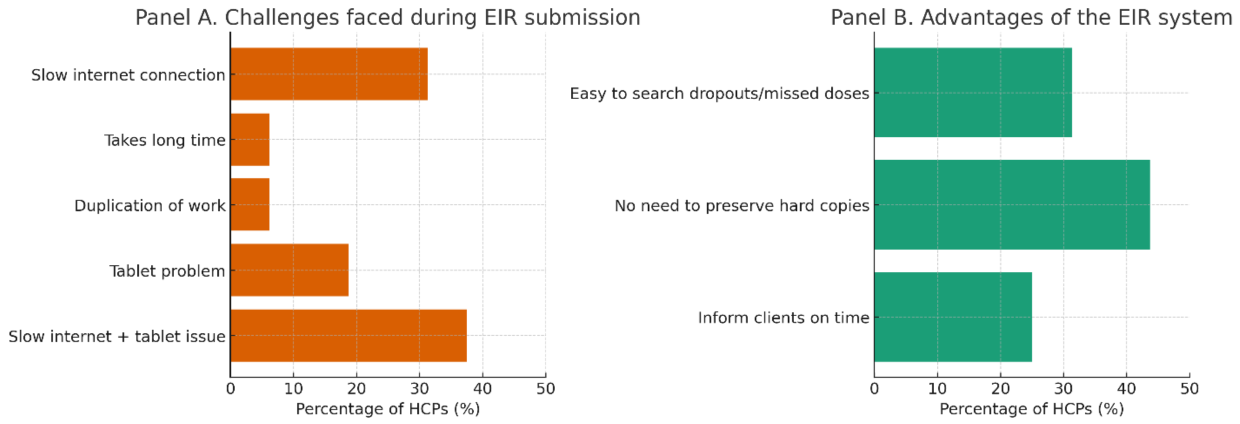

Figure 1. Challenges and advantages of the EIR system reported by HCPs (n=16). Panel A shows the percentage of providers reporting specific challenges during EIR submission, while Panel B highlights the key advantages of using the EIR platform.

Discussion

Summary of the findings

The EIR in RCC was well integrated into routine immunization services. Most registrations were completed at EPI centers, supported by outreach activities. Vaccination coverage remained high, and all eligible children were successfully registered. Both caregivers and HCPs reported high satisfaction, citing ease of use, SMS reminders, and improved record management. Despite occasional technical challenges, the EIR was widely accepted and showed strong potential for broader scale-up.

Strengths and limitations

This study provides early assessment of EIR feasibility in Bangladesh, offering insights to support potential national scale-up. By combining quantitative data from caregivers and HCPs with qualitative insights from open-ended responses, the study provides a comprehensive understanding of the EIR's usability, user satisfaction, and perceived barriers and enablers. The inclusion of multiple respondent groups and a complete dataset covering 321 participants strengthens the validity of the findings. Furthermore, this research addresses an important evidence gap and offers policy-relevant insights that can inform digital health strategies and immunization program planning in LMICs.

However, several limitations should be considered when interpreting the results. First, the study was conducted in a single urban pilot site within RCC, which may limit the generalizability of the findings to rural or resource-constrained areas with different infrastructural realities. Second, the cross-sectional design captured only a snapshot in time and did not allow assessment of the long-term impact of EIR implementation on

vaccine coverage, timeliness, or dropout reduction. While purposive sampling ensured the inclusion of participants directly involved with the EIR, it may introduce selection bias. Additionally, reliance on self-reported data for satisfaction levels, perceived challenges, and advantages raised the possibility of recall and social desirability biases. Finally, as the EIR pilot received technical and infrastructural support from WHO and local authorities, system performance may differ in settings with limited resources, weaker internet connectivity, or fewer trained staff. The absence of a comparison group using traditional paper-based registries also restricts the ability to attribute all observed improvements solely to the EIR system.

Interpretation in the light of published papers

The observed registration timeliness of 88% within 45 days is noteworthy. Similar findings have been reported in Tanzania, Zambia, and Vietnam, where EIRs significantly enhanced early registration and follow-up [18]. SMS reminders, adopted in multiple LMIC settings, further strengthen adherence to vaccination schedules by reducing missed appointments and improving completion rates [19-21]. The high caregiver satisfaction (91%) observed in our study is consistent with evidence from Kenya and Pakistan, where registries were associated with better user engagement and perceived service quality [22, 23].

Despite these achievements, several challenges persist. Technological barriers, including intermittent internet access, device limitations, and software downtime, were reported by providers. These are consistent with findings from Zambia and Pakistan, where inadequate infrastructure occasionally limited EIR functionality [23, 24]. Addressing these

issues will require sustained investment in digital infrastructure, offline-ready solutions, and refresher training for frontline staff.

Globally, studies demonstrate that EIRs support efficient resource allocation, enable proactive identification of zero-dose children, and facilitate integration with logistics management systems [25]. In Vietnam, the National Immunization Information System successfully scaled nationally by leveraging phased rollouts and partnerships across government, donors, and the private sector [26]. Similarly, the Better Immunization Data' initiative in Tanzania and Zambia demonstrated how EIRs could be deployed alongside stock-tracking systems, reducing duplication and improving operational efficiency [24]. These experiences suggest a scalable path for Bangladesh, particularly if RCC's lessons are integrated into national rollout strategies.

With Bangladesh's immunization agenda prioritizing equity and timeliness, EIRs offer an opportunity to improve both service delivery and monitoring. However, nationwide implementation will require coordinated investment in improving internet connectivity and speed, capacity building, and integration with broader health information systems. Leveraging EIR data for microplanning, outreach, and resource allocation could further reduce missed opportunities for vaccination and improve health outcomes.

Conclusion

The EIR pilot in RCC was promising and contributed to improved registration timeliness, vaccination coverage, and service delivery. Despite minor technical challenges, its broad acceptance indicates high potential for nationwide scale-up to strengthen immunization monitoring and advance digital health in Bangladesh.

Author Contributions

MJH drafted the first version of the manuscript with support from AKMK, RB, CD, MTHS, NU, and AFMSK. AKMK, RB, and CD led the conceptualization, while MJH and MTHS conducted the analysis. All authors critically reviewed and approved the final version of the manuscript.

Data availability

All relevant data are within the manuscript.

Funding

The authors received no specific funding for this work.

Competing Interests

The authors have declared that no competing interests exist.

Acknowledgements

We are grateful to WHO-IVD, Bangladesh for their funding support to carry out the evaluation work. We sincerely thank the study participants and the dedicated RCC and WHO staff for their invaluable support throughout this study.

References

1. UNICEF. Immunization (July 2025). https://data.unicef.org/topic/child-health/immunization/. Last accessed: 17 November 2025.
2. Carter A, Msemburi W, Sim SY, Gaythorpe KA, Lambach P, Lindstrand A, et al. Modeling the impact of vaccination for the immunization Agenda 2030: Deaths averted due to vaccination against 14 pathogens in 194 countries from 2021 to 2030. Vaccine. 2024;42:S28-S37.
3. Wariri O, Okomo U, Kwarshak YK, Utazi CE, Murray K, Grundy C, et al. Timeliness of routine childhood vaccination in 103 low-and middle-income countries, 1978–2021: A scoping review to map measurement and methodological gaps. PLOS global public health. 2022;2(7):e0000325.
4. Haeuser E, Byrne S, Nguyen J, Raggi C, McLaughlin SA, Bisignano C, et al. Global, regional, and national trends in routine childhood vaccination coverage from 1980 to 2023 with forecasts to 2030: a systematic analysis for the Global Burden of Disease Study 2023. The Lancet. 2025.
5. World Health Organization. Immunization Agenda 2030: A Global Strategy to Leave No One Behind. https://www.who.int/teams/immunization-vaccines-and-biologicals/strategies/ia2030. Last accessed: 17 November 2025.
6. Sarkar PK, kumar Sarker N, Doulah S, Bari TIA. Expanded programme on immunization in Bangladesh: a success story. Bangladesh Journal of Child Health. 2015;39(2):93-8.
7. Kundu S, Kundu S, Seidu A-A, Okyere J, Ghosh S, Hossain A, et al. Factors influencing and changes in childhood vaccination coverage over time in Bangladesh: a multilevel mixed-effects analysis. BMC Public Health. 2023;23(1):862.
8. Directorate General of Health Services, Bangladesh. Coverage Evaluation Survey 2023. https://www.sdg.gov.bd/uploads/indicator-data/EPI%20CES%202023%20-%20First%2028%20pages.pdf. Last accessed: 17 November 2025.
9. Mantel C, Hugo C, Federici C, Sano N, Camara S, Rodriguez E, et al. Impact of electronic immunization registries and electronic logistics management information systems in four low- and middle-income countries: Guinea, Honduras, Rwanda, and Tanzania. Vaccine. 2025;54:127066.
10. Mc Kenna P, Broadfield LA, Willems A, Masyn S, Pattery T, Draghia-Akli R. Digital health technology used in emergency large-scale vaccination campaigns in low-and middle-income countries: a narrative review for improved pandemic preparedness. Expert Review of Vaccines. 2023;22(1):243-55.
11. Zarekar M, Al-Shehabi H, Dörner R, Weishaar H, Lennemann T, El Bcheraoui C, et al. The impact of information and communication technology on immunisation and immunisation programmes in low-income and middle-income countries: a systematic review and meta-analysis. EBioMedicine. 2025;111.
12. Nguyen NT, Vu HM, Dao SD, Tran HT, Nguyen TXC. Digital immunization registry: evidence for the impact of mHealth on enhancing the immunization system and improving immunization coverage for children under one year old in Vietnam. Mhealth. 2017;3:26.
13. 13. Mvundura M, Di Giorgio L, Vodicka E, Kindoli R, Zulu C. Assessing the incremental costs and savings of introducing electronic immunization registries and stock management systems: evidence from the better immunization data initiative in Tanzania and Zambia. The Pan African Medical Journal. 2020;35(Suppl 1):11.
14. Chandir S, Siddiqi DA, Dharma VK, Shah MT, Turab A, Khan MI, et al. Zindagi Mehfooz (safe life) digital immunization registry: Leveraging low-cost technology to improve immunization coverage and timeliness in Pakistan. Iproceedings. 2018;4(2):e11770.

15. Dolan SB, Carnahan E, Shearer JC, Beylerian EN, Thompson J, Gilbert SS, et al. Redefining vaccination coverage and timeliness measures using electronic immunization registry data in low-and middle-income countries. Vaccine. 2019;37(13):1859-67.
16. World Health Organization. Successful implementation of Electronic Immunization registration in Rajshahi. https://www.who.int/bangladesh/news/detail/13-10-2022-successful-implementation-of-electronic-immunization-registration-in-rajshahi. Last accessed: 17 Novemeber 2025.
17. Wagner AL. The use and significance of vaccination cards. Hum Vaccin Immunother. 2019;15(12):2844-6.
18. Carnahan E, Nguyen L, Dao S, Bwakya M, Mtenga H, Duong H, et al. Design, development, and deployment of an electronic immunization registry: experiences from Vietnam, Tanzania, and Zambia. Global Health: Science and Practice. 2023;11(1).
19. Oliver-Williams C, Brown E, Devereux S, Fairhead C, Holeman I. Using mobile phones to improve vaccination uptake in 21 low-and middle-income countries: systematic review. JMIR mHealth and uHealth. 2017;5(10):e7792.
20. Eze P, Lawani LO, Acharya Y. Short message service (SMS) reminders for childhood immunisation in low-income and middle-income countries: a systematic review and meta-analysis. BMJ Glob Health. 2021;6(7).
21. Yunusa U, Garba SN, Umar AB, Idris SH, Bello UL, Abdulrashid I, et al. Mobile phone reminders for enhancing uptake, completeness and timeliness of routine childhood immunization in low and middle income countries: a systematic review and meta-analysis. Vaccine. 2021;39(2):209-21.
22. Juma S, Tabu C, Gura Z, Waweru S, Njeru I. User Acceptability of Electronic Vaccine Registry Created using Simple Mobile Phone Technology in Nyandarua County, Kenya. 2022.
23. Mechael P, Gilani S, Ahmad A, LeFevre A, Mohan D, Memon A, et al. Evaluating the “Zindagi Mehfooz” electronic immunization registry and suite of digital health interventions to improve the coverage and timeliness of immunization services in Sindh, Pakistan: mixed methods study. J Med Internet Res. 2024;26:e52792.
24. Dolan SB, Alao ME, Mwansa FD, Lymo DC, Bulula N, Carnahan E, et al. Perceptions of factors influencing the introduction and adoption of electronic immunization registries in Tanzania and Zambia: a mixed methods study. Implementation Science Communications. 2020;1(1):38.
25. Ingle EA, Shrestha P, Seth A, Lalika MS, Azie JI, Patel RC. Interventions to vaccinate zero-dose children: a narrative review and synthesis. Viruses. 2023;15(10):2092.
26. Dang H, Dao S, Carnahan E, Kawakyu N, Duong H, Nguyen T, et al. Determinants of scale-up from a small pilot to a national electronic immunization registry in Vietnam: qualitative evaluation. J Med Internet Res. 2020;22(9):e19923.
