# Supplementary material for: Feasibility and acceptability of an electronic immunization registry in urban Bangladesh
Source: PLoS One. 2026 Jul 2;21(7):e0352972. doi: 10.1371/journal.pone.0352972 (PMC13327150; doi:10.1371/journal.pone.0352972)
Supplement: Attachment — Submitted filename: Review Report_PONE-D-25-67616 .docx [file pone.0352972.s004.docx]

**Review Report_ PLOS One**

**Manuscript:** Feasibility and acceptability of an electronic immunization registry in urban Bangladesh

**Authors**: Md. Jawadul Haque, A K M Kamruzzaman, Rajendra Bohara, Chiranjit Das, Md., Tarjumanul Haque Sanij, Abul Fazal Md., Shahabuddin Khan, F A M Anjuman Ara Begum, Nazim Uzzaman

**Recommendation: MAJOR REVISION**

**General comments:**

This study is very important, as it seeks to provide evidence on the feasibility, acceptability, challenges and advantages of an EIR in Bangladesh. This can provide evidence for a national scale-up, and other LMICs can also benefit from this experience in Bangladesh.

That said, I believe the manuscript can benefit from some revisions in the focus and analysis of the results to make it more robust.

**Specific Comments:**

1. Consider revising your topic to reflect the content of the study. Most of the data reported in the manuscript speaks to caregiver and HCP satisfaction, not the feasibility of the EIR.
2. I recommend that the authors consider reporting this study in line with at least one of the WHO digital health evaluation frameworks. This will make it easier to compare with other studies in different settings in the LMICs.
3. I recommend that the authors clarify whether they are measuring the feasibility or satisfaction or both. Such clarity would help to focus on the substance of the study. Starting from the title of the study, through the results and discussions, ensure consistency.
4. Report the Cronbach's alpha (internal consistency) of the Likert scale here (Lines 101-103).
5. Conduct some advanced analytical statistics to compare the satisfaction between caregivers and HCPs (Line 106).
6. In line 107, you stated that you computed means and standard deviations. However, there are no means and SDs reported in your results. Include them for the satisfaction of caregivers and HCPs, respectively.
7. Insert the Ethics approval number here (111-112).
8. I would recommend that the authors conduct a more advanced analysis of the data, comparing the HCP satisfaction and Caregiver satisfaction. Use an " Independent t-test (caregivers vs providers)" or any other advanced statistical method.
9. After all these revisions, the discussion and conclusion have to be adapted to the results.
