# Supplementary material for: Feasibility and acceptability of an electronic immunization registry in urban Bangladesh
Source: PLoS One. 2026 Jul 2;21(7):e0352972. doi: 10.1371/journal.pone.0352972 (PMC13327150; doi:10.1371/journal.pone.0352972)
Supplement: Attachment — Submitted filename: PLOS ONE_PONE-D-25-67616.docx [file pone.0352972.s002.docx]

**REVIEWER’S COMMENT FOR THE MANUSCRIPT NO. PONE-D-25-67616**

**REVIEWER: SURAJO ADAMU WADA, PhD**

| **SECTION** | **COMMENT** |
| --- | --- |
| **TITLE** | It is concise and appropriately represent the manuscript content |
| **ABSTRACT** | The abstract reflects the manuscript content. |
| Authors affiliations | These should be reviewed to follow the guidelines of the journal. |
| **INTRODUCTION** | The introduction is fairly written |
| **MATERIALS AND METHODS** |  |
| Study participants | Since the previous prevalence of similar studies was not available, the sample size should   1. Be calculated using a prevalence value of 0.5. 2. Use other methods for sample size determination, such as the sample size table or the online sample size calculator. 3. Otherwise, provide a strong reason for using the caregivers that lack vaccination. |
| Ethics | This sub-section is supposed to come before data collection.  The number of the ethical approval should be included (if any). |
| **RESULTS** |  |
| Table 1 | The authors are expected to give reason (s) why HCPs are not included, and the target achieved was 100% |
| Perceived challenges and benefits | Paragraph 1. Line 150 – 154: the figure stated is missing, and if present, should be after Figure 1.  The result section has to be updated. |
| Figure 1 | The resolutions for the figure labells should be improved for clarity. |
| **DISCUSSION** | This is advised to follow the exact guidelines of the journal. |
| **CONCLUSION** | Well written |
| **REFERENCE** | All references in the text have to appear in the reference list at the end of the manuscript |
| **TYPE SETTING** | The paragraphs are to be indented to the right, except for the first paragraph in a section or subsection.  Sentences should not start with abbreviations as in line 150. |
| **MANUSCRIPT STRUCTURE** | The authors are advised to visit the journal website for proper structuring of the article. |
| **ENGLISH EDITING** | It will be better for the authors to subject the article to English editing services. |
| **MY RECOMMENDATION** | The article is recommended to be accepted for publication if these updates are made accordingly. |
