# Supplementary material for: Feasibility and acceptability of an electronic immunization registry in urban Bangladesh
Source: PLoS One. 2026 Jul 2;21(7):e0352972. doi: 10.1371/journal.pone.0352972 (PMC13327150; doi:10.1371/journal.pone.0352972)
Supplement: Attachment — Submitted filename: Review-Plos One.docx [file pone.0352972.s003.docx]

Materials and Methods

- As there is no previous study, it is recommended to calculate as 50%. But this study calculates 16% which is based on caregivers who are not vaccinated. If I suggest to calculate the sample size again with 50% as prevalence rate.
- Data analysis section is lack of proper rigorous analysis.
- Ethical approval was taken but there is no approval ID.
- Lack of description about data collection process, using tools, design questionnaire, validation of questionnaire, any pilot survey before collection of data and detailed information about this entire process.

Results

- I would like to suggest authors to add a demographic table for this with 95% CI
- How target achieved was calculated as sample was 207. Similarly, methodology said their sample size was 207 but they collected 321 data. There is a lack of proper validation for this.
- Table 2 mentioned 5-level likert but there is missing other two.
