# Supplementary material for: Feasibility and acceptability of an electronic immunization registry in urban Bangladesh
Source: PLoS One. 2026 Jul 2;21(7):e0352972. doi: 10.1371/journal.pone.0352972 (PMC13327150; doi:10.1371/journal.pone.0352972)
Supplement: Attachment — Submitted filename: Response_to_Reviewers.docx [file pone.0352972.s007.docx]

16^th^ May 2026

Dr Emily Chenette

Editor-in-Chief
PLOS ONE

**Re: PONE-D-25-67616R1: Feasibility and acceptability of an electronic immunization registry in urban Bangladesh**

Dear Dr Chenette,

Thank you for your interest in our paper and for enclosing the encouraging and helpful comments from your reviewers. We appreciate the time and effort taken by the reviewers and the editorial team to read our manuscript and provide constructive comments to improve it. We have pleasure in submitting our revised manuscript entitled “Feasibility and acceptability of an electronic immunization registry in urban Bangladesh” to be considered for publication in PLOS ONE.

For your convenience, we have reproduced the editor’s and reviewers’ comments verbatim, before detailing our response to each of the points made. Page and line numbers refer to the ‘Revised Manuscript with Track Changes’.

**Academic Editor: Dr Samuel Kofi Tchum**

Thank you for submitting your manuscript to PLOS ONE. After careful consideration, we feel that it has merit but does not fully meet PLOS ONE’s publication criteria as it currently stands. Therefore, we invite you to submit a revised version of the manuscript that addresses the points raised during the review process.

Thank you for your encouraging words and for enclosing the helpful comments from your reviewers. We have responded to the reviewers’ comments in details below.

**Reviewer #1**

1. Generalizability: The study is limited to a single urban pilot site (Rajshahi City Corporation). Findings may not reflect rural or resource-constrained areas. Please expand the discussion on how infrastructural differences (e.g., internet connectivity, staffing) might affect nationwide rollout.

Thank you for this comment. We agree and have expanded the limitations and implementation discussion to emphasize that findings from a single urban pilot setting may not be transferable to rural or resource-constrained settings where infrastructure, staffing, and connectivity differ. We also added that nationwide implementation would require investment in internet connectivity, capacity building, and integration with broader health information systems (page 17, lines 322–328; page 19, lines 352–365; page 19, lines 375–382).

2. Study Design: The cross-sectional design provides only a snapshot in time. Longitudinal or comparative studies would be needed to assess sustainability, dropout reduction, and equity impacts. Please acknowledge this limitation more explicitly.

Thank you for this comment. We have now explicitly acknowledged this limitation. We also revised the Abstract, Methods, Discussion, and Conclusion to state that the study was not designed to evaluate effectiveness or causal impact (page 2, lines 31–40; page 7, lines 114–123; page 17, lines 313–328; page 20, lines 383–388).

3. Sampling Bias: Purposive sampling may have introduced bias, particularly if participants were more likely to be favorable toward the EIR. Clarification on participant selection criteria would strengthen the methodology.

Thank you. We agree that purposive sampling may have introduced selection bias, particularly because participants were recruited from individuals already exposed to EIR-supported services and may therefore have been more likely to report positive perceptions. We have revised the Methods section to more clearly describe the participant selection approach and rationale for using purposive sampling in this descriptive operational assessment. We have also expanded the limitations section to acknowledge that this approach may have overestimated satisfaction and perceived benefits among relatively engaged users (page 8, lines 125–142; page 17, lines 317–320).

4. Self-reported Satisfaction: Satisfaction measures rely on self-report, which may be subject to recall and social desirability bias. Please discuss this limitation in more depth and consider triangulating with objective usage data in future studies.

Thank you for this comment. We agree that self-reported satisfaction may be affected by recall and social desirability bias. We have expanded the limitations section to acknowledge this issue and have noted that future studies should include objective data sources, such as registry audits or direct observation of system use (page 17, lines 319–328).

5. Absence of Control Group: Without comparison to paper-based registries, it is difficult to attribute improvements solely to the EIR. Acknowledging this limitation would improve transparency.

Thank you for this comment. We agree that, without a comparator, observed outcomes cannot be attributed solely to the EIR. We have revised the manuscript to avoid causal language and clarify that the study provides descriptive findings (page 7, lines 114–123; page 17, lines 315–318; page 20, lines 384–388).

6. Technical Challenges: While slow internet and device malfunctions are noted, the discussion could be expanded to include potential solutions (e.g., offline-ready systems, infrastructure investment, refresher training). This would strengthen the scalability argument.

Thank you for this suggestion. We have now expanded the Discussion to include mitigation strategies, including digital infrastructure investment, offline-capable systems, and refresher training for frontline staff (page 19, lines 352–365).

7. Data Availability: The statement “all relevant data are within the manuscript” may limit reproducibility. Consider depositing anonymized datasets in a public repository to align with open science practices.

Thank you. We have now updated the Data availability statement to clarify that the de-identified dataset supporting the findings is provided as a supplementary file (page 21, lines 400–404).

8. Clarity of Writing: Some sections of the discussion repeat global evidence without fully connecting back to the Bangladesh context. Strengthening the local interpretation would enhance impact.

Thank you. We have revised the Discussion to reduce repetition of global evidence and to connect the findings to the Bangladesh implementation context, including infrastructure, workforce capacity, system integration, and phased implementation considerations (page 18, lines 342–351; page 19, lines 352–382).

9. Figures and Tables: Figures summarizing caregiver and HCP satisfaction could benefit from clearer legends and consistent formatting of percentages.

Thank you for this suggestion. Upon further consideration, we removed the figure because the number of HCPs was small, and percentage-based visualization could be misleading. The findings are now presented descriptively in the Results, and Tables 2 and 3 now include all five Likert response categories, including zero-response categories (page 12, lines 226–230; page 13, lines 247–259).

10. Methodological Detail: Please provide more information on questionnaire validation and how qualitative response s were coded.

Thank you. We have expanded the Methods section to describe questionnaire pretesting and clarified that open-ended responses were not subjected to formal qualitative coding but were reviewed and descriptively summarized to identify commonly reported issues and experiences (page 8, lines 143–157; page 9, lines 158–168).

11. Terminology Consistency: Ensure consistent use of abbreviations (e.g., EIR, EPI) throughout the manuscript.

Thank you. We have reviewed the manuscript and standardized abbreviations, including EIR, EPI, RCC, LMICs, and HCPs, throughout the manuscript.

12. Ethical and Publication Considerations: Ethical approval and informed consent are clearly stated; no concerns identified.

Thank you. We also have strengthened the Ethics section by adding the ethics approval reference number and data confidentiality procedures (page 9, lines 170–175).

13. No evidence of dual publication or ethical violations.

Thank you for confirming this.

14. Financial disclosure and competing interests are appropriately declared.

Thank you.

15. Overall Recommendation: This manuscript makes a meaningful contribution to the literature on digital health and immunization in LMICs. With revisions to strengthen methodological transparency, expand discussion of limitations, and improve data accessibility, the paper will be well-positioned for publication.

Thank you for this encouraging recommendation. We have revised the manuscript to strengthen methodological transparency, expand limitations, avoid causal overinterpretation, improve reporting of Likert-scale data, and update the Data availability statement.

**Reviewer #2**

16. This manuscript presents a cross-sectional assessment of the feasibility and acceptability of an electronic immunization registry (EIR) in urban Bangladesh. The topic is relevant, the study appears technically sound, and the conclusions are generally supported by the data. However, several points require clarification.

Thank you for your comment. We have addressed each clarification point below.

17. Funding statement: There is an inconsistency between the Funding and Acknowledgements sections. The authors should clarify whether WHO-IVD support was financial or in-kind and ensure consistency between these sections.

Thank you for this comment. In response to the journal’s technical check following the revised submission, funding-related text has now been removed from the manuscript file, including the Funding, Competing Interests, and Acknowledgements sections. The Acknowledgements section in the revised manuscript now reads: “We thank the study participants for their contribution to this study.”

18. Data availability: For a survey-based study, PLOS data policy typically requires availability of the underlying de-identified dataset. The authors should clarify whether the raw survey data can be shared and update the Data Availability statement accordingly.

Thank you. We have updated the Data availability statement to indicate that the de-identified dataset is available as a supplementary file (page 21, lines 400–404)

19. The description of the Likert scale is inconsistent (described as 5-point but defined from 0 to 5) and should be corrected.

Thank you for identifying this error. We have now corrected the Likert-scale description throughout the manuscript. The scale is now consistently described as a 5-point scale ranging from 1 to 5 (page 8, lines 151–157; page 12, lines 227–230; page 14, lines 249–252).

20. Satisfaction outcomes are reported without explicit dissatisfied categories; the authors should clarify how these were handled and how overall satisfaction was calculated.
Thank you for this comment. We have revised Tables 2 and 3 to display all five Likert categories, including categories with zero responses. We also clarified that Likert items were analyzed individually and were not combined into a composite or overall satisfaction score (page 9, lines 159–168; page 12, lines 226–230; page 13–14, lines 247–252).

21. Minor typographical and formatting issues should be addressed.

Thank you. We have reviewed the manuscript and corrected typographical, grammatical, and formatting issues.

**Reviewer #3**

22. Study design and causal interpretation: The cross-sectional design of the study limits the ability to attribute the observed high vaccination coverage and improved registration timeliness directly to the electronic immunization registry. Several statements in the Abstract, Results, and Conclusions sections appear to imply an impact of the EIR rather than focusing strictly on feasibility and acceptability. The conclusions should be reframed to clearly distinguish association from causation and to avoid over-claiming system effectiveness.

Thank you. We agree and have revised the Abstract, Methods, Results, Discussion, and Conclusion to remove causal language and to frame the findings as descriptive perceptions and reported operational experiences rather than evidence of effectiveness or system-level impact (page 2–3, lines 31–58; page 7, lines 114–123; page 15–16, lines 277–295; page 20, lines 383–388).

23. Sampling strategy and potential bias: Caregivers were selected using purposive sampling, and all participants were already engaged with the EIR system. This approach introduces selection bias and likely inflates estimates of satisfaction and vaccination coverage. The limitations section should be expanded to explicitly discuss how purposive sampling and the exclusion of non-users may have influenced the findings.

Thank you. We have now clarified the purposive sampling strategy in the Methods and explicitly acknowledged selection bias in the limitations (page 8, lines 136–142; page 17, lines 317–320)

24. Vaccination coverage results (100% coverage): The reporting of 100% vaccination coverage for multiple antigens in an urban setting is unusual and requires careful contextualization. It is unclear whether the reported coverage reflects true population-level coverage or only coverage among children registered in the EIR system. The authors should clarify the denominators used, explicitly state that coverage estimates apply only to registered children, and temper interpretations accordingly.

We appreciate your concern and agree that the coverage results require careful contextualization. We have now clarified that these estimates reflect reported vaccination status among children registered in the EIR system and should not be interpreted as population-level vaccination coverage. We also have revised the Results and Discussion to avoid population-level interpretation (page 10, lines 183–190; page 11, lines 194–203).

25. Very small healthcare provider sample size: Only 16 healthcare providers were included in the study, yet results are presented in percentages and used to support relatively strong conclusions. This small sample size limits both statistical reliability and interpretive robustness. The manuscript should more explicitly acknowledge this limitation and avoid broad generalizations based on these data.

Thank you. We agree and now explicitly state that the HCP sample was small and that findings should be interpreted as indicative of provider perceptions within this setting. We have also removed broad claims based on HCP percentages (page 13, lines 231–242; page 17, lines 321–322)

26. Data availability statement: The manuscript states that all relevant data are contained within the manuscript, yet the results appear to be derived from underlying survey datasets. This may not fully align with PLOS ONE data transparency requirements. The authors should clarify whether anonymized datasets can be shared as supplementary files or deposited in a public repository.
Thank you. We have revised the Data availability statement to indicate that the de-identified dataset is available as a supplementary file (page 21, lines 400–404)

27. Likert scale description: The Likert scale is described in the Methods section as ranging from 0 to 5, while the tables appear to reflect a 1 to 5 scale. This discrepancy should be clarified and standardized throughout the manuscript.

Thank you for identifying this error. We have now corrected the Methods and table footnotes to describe the 5-point Likert scale consistently as 1 to 5 (page 8, lines 151–157; page 12, lines 227–230; page 14, lines 249–252).

28. Figures and tables: There are inconsistencies in figure numbering in the text, such as references to Figure 1 and Figure 3. These should be checked and corrected. In addition, figure captions should be improved to be fully self-explanatory without requiring reference to the main text.

Thank you for this suggestion. We have checked figure numbering and removed the figure from the manuscript, as it added limited analytical value and could be misleading given the small number of HCPs. The relevant findings are now presented descriptively in the Results section (page 14, lines 254–259).

**Reviewer #4**

29. Overall Assessment: This manuscript presents a cross-sectional evaluation of the feasibility and acceptability of an Electronic Immunization Registry (EIR) piloted in an urban setting in Bangladesh. The topic is timely and relevant, particularly in the context of digital health scale-up in low- and middle-income countries (LMICs) aligned with Immunization Agenda 2030. The study contributes descriptive evidence on user satisfaction, registration timeliness, and operational challenges from both caregivers and healthcare providers. While the findings are generally positive and policy-relevant, limitations in study design, sampling strategy, and inferential depth constrain the strength of conclusions regarding effectiveness and scalability. With substantive revisions to clarify methodology, contextualize findings, and temper conclusions, the manuscript could be suitable for publication.

Thank you for this constructive overall assessment. We have now revised the manuscript to clarify methodology, contextualize findings, and temper conclusions related to effectiveness and scalability.

30. Study design limits causal inference and claims of improvement: The cross-sectional design without a comparator (e.g., pre-EIR baseline or paper-based registry group) limits the ability to attribute high vaccination coverage, registration timeliness, or satisfaction directly to the EIR. Statements suggesting that the EIR “improved” coverage or service delivery should be reframed more cautiously to reflect association rather than impact.

Thank you. We have now revised the manuscript to make clear that the study was a descriptive assessment and was not intended to evaluate effectiveness or causal impact. We have removed or revised wording that implied improvement, impact, or scalability as established outcomes (page 2–3, lines 31–58; page 7, lines 114–123; page 20, lines 383–388).

31. Sampling strategy and representativeness require clearer justification: The use of purposive sampling to recruit “information-rich” caregivers and inclusion of all HCPs involved in the EIR introduces potential selection bias. The manuscript should more explicitly discuss how this approach may overestimate satisfaction and feasibility, and how findings may differ among less-engaged users or marginalized populations.

Thank you. We have clarified the purposive sampling strategy in the Methods section and acknowledged that recruiting participants already exposed to EIR-supported services may have overestimated satisfaction and perceived benefits among relatively engaged users. We have also expanded the limitations section to note that the findings may differ among less-engaged or underserved populations and may not be fully transferable to rural or resource-constrained settings (page 8, lines 136–142; page 17, lines 317–328)

32. Interpretation of vaccination coverage results may be overstated: Reported vaccination coverage rates (100% for several antigens) are exceptionally high and may reflect programmatic selection rather than population-level performance. The manuscript should clarify whether these figures represent verified coverage within the EIR cohort only and should avoid implying general population coverage improvements.

Thank you. We have now clarified that vaccination figures apply only to children registered in the EIR study sample and should not be interpreted as population-level coverage estimates (page 10, lines 183–190; page 11, lines 194–203).

33. Limited analytical depth beyond descriptive statistics: The analysis is restricted to descriptive statistics, which is appropriate for feasibility assessment but limits insight into factors associated with satisfaction, delayed registration, or reported challenges. The authors should either justify this limitation explicitly or explore simple stratified analyses (e.g., caregiver demographics vs. satisfaction) if data permit.

Thank you for this comment. Given the descriptive and exploratory nature of the study, and the small number of HCPs, we did not conduct inferential or stratified analyses. We have now clarified this in the Methods (page 9, lines 158–168).

34. Clarify Likert scale description and reporting: The Likert scale is described as ranging from 0 to 5, but tables appear to reflect only categorical responses without explicit mention of scale anchors. Consistency and clarity in scale description would improve transparency.

Thank you. We have now corrected the Likert-scale description throughout and specified the 1–5 response anchors in the Methods and table footnotes (page 8, lines 151–157; page 12, lines 227–230; page 14, lines 249–252).

35. Improve clarity in tables and figures: Tables 2 and 3 would benefit from clearer denominators and explicit reporting of missing or neutral responses. Figure captions should specify whether percentages are mutually exclusive or allow multiple responses.

Thank you for this suggestion. Tables 2 and 3 now report all five Likert categories, including zero-response categories, and include denominator footnotes. We removed the figure to avoid overinterpretation based on the small HCP sample (page 12, lines 226–230; page 13–14, lines 247–252).

36. Recommendation: The study is relevant and methodologically sound for a feasibility assessment, but substantial revisions are needed to address overinterpretation of results, clarify sampling limitations, and align conclusions more closely with the descriptive nature of the data.

Thank you for this constructive recommendation. We have now revised the manuscript to address overinterpretation, clarify sampling limitations, and align conclusions with the descriptive nature of the data.

**Reviewer #5**

37. This is very good research for the Bangladeshi population. This type of research should be performed. If this article publishes in national journal, then the effectiveness will be more for the national population. Thank you.

Thank you for this encouraging comment. We have further strengthened the Bangladesh-specific context while keeping the interpretation appropriately cautious and descriptive (page 5–6, lines 74–107; page 19, lines 375–382).

**Reviewer #6**

38. The authors are advised to visit the journal website for proper structuring of the article. The article is recommended to be accepted for publication if updated accordingly. The review is uploaded as an attachment.

Thank you for this comment. We have reviewed the journal requirements and revised the manuscript structure, formatting, and file preparation accordingly.

39. Title: It is concise and appropriately represent the manuscript content.

Thank you.

40. Abstract: The abstract reflects the manuscript content.

Thank you. We have further revised the Abstract to align with the descriptive framing of the study (page 2–3, lines 18–58).

40.1 Authors affiliations: These should be reviewed to follow the guidelines of the journal

Thank you. We have reviewed and formatted the author affiliations according to journal requirements (page 1, lines 1–14).

41. Introduction: The introduction is fairly written.

Thank you. We have further revised the Introduction to provide clearer context and better alignment with the study aim (page 5–6, lines 66–107).

Materials and methods:

42 Study participants: Since the previous prevalence of similar studies was not available, the sample size should 1) be calculated using a prevalence value of 0.5; 2) use other methods for sample size determination, such as the sample size table or the online sample size calculator; 3) otherwise, provide a strong reason for using the caregivers that lack vaccination.

We acknowledge this concern. We have now revised the Methods to clarify that the initial sample size estimate was used to guide recruitment and was not intended to support statistical inference. We have also clarified that purposive sampling was used because the study was descriptive and operational, and participants needed direct exposure to the RCC EIR (page 7–8, lines 124–142).

43. Ethics: This sub-section is supposed to come before data collection. The number of the ethical approval should be included (if any).

Thank you. We have now included the ethics approval reference number and added data confidentiality procedures (page 9, lines 170–175).

Results

44. Table 1: The authors are expected to give reason (s) why HCPs are not included, and the target achieved was 100%

Thank you for this comment. We have now clarified that Table 1 reports registration and vaccination-related indicators for children/caregivers, whereas HCP findings are reported separately in the satisfaction and challenges sections. We also clarified that these estimates apply only to children registered in the EIR study sample and are not population-level coverage estimates (page 10–11, lines 183–203).

45. Perceived challenges and benefits: Paragraph 1. Line 150 – 154: the figure stated is missing, and if present, should be after Figure 1. The result section has to be updated.

Thank you for identifying this. Figure 3’ was a typo. We have now checked figure numbering and removed the figure from the manuscript, as it added limited analytical value and could be misleading given the small number of HCPs. The relevant findings are now presented descriptively in the Results section (page 14, lines 254–259).

46. Figure 1: The resolutions for the figure labels should be improved for clarity.

Thank you. We have now removed the figure because it added limited analytical value and could be misleading given the small HCP sample. Findings are now reported descriptively in the Results.

47. Discussion: This is advised to follow the exact guidelines of the journal.

Thank you. We have revised the Discussion to better align with journal expectations and to focus on interpretation, limitations, and implications.

48. Conclusion: Well written

Thank you. We have further revised the Conclusion to ensure that it aligns well with descriptive nature of the study (page 20, lines 383–388).

49. Reference: All references in the text have to appear in the reference list at the end of the manuscript

Thank you. We have checked the in-text citations and reference list for consistency.

50. Type setting: The paragraphs are to be indented to the right, except for the first paragraph in a section or subsection. Sentences should not start with abbreviations as in line 150.

Thank you. We have revised the manuscript formatting according to journal guidelines and corrected sentences beginning with abbreviations where applicable.

51. Manuscript structure: The authors are advised to visit the journal website for proper structuring of the article.

Thank you. We have revised the manuscript structure according to the journal requirements.

52. English editing: It will be better for the authors to subject the article to English editing services.

Thank you. We have carefully edited the manuscript to improve clarity, grammar, and readability.

53. My recommendation: The article is recommended to be accepted for publication if these updates are made accordingly.

Thank you for the positive recommendation. We have addressed the requested updates throughout the manuscript.

**Reviewer #7**

54. The manuscript presents a technically sound feasibility study, with an appropriate study design and descriptive analyses that are aligned with the stated objectives. The data supports the conclusions regarding feasibility and acceptability of the EIR among caregivers and healthcare providers, and the authors appropriately acknowledge key methodological limitations. However, the manuscript would benefit from a clearer description of how caregivers interact with the EIR system, as caregivers appear to be largely passive recipients of EIR outputs rather than active users, which should be more clearly reflected in the interpretation of caregiver-related findings.
Thank you for this insightful observation. We agree and now have clarified in the Methods, Results, and Discussion that caregivers were recipients of EIR-supported services, whereas HCPs were direct users of the digital platform (page 7, lines 120–123; page 11, lines 207–216; page 16, lines 290–295).

55. Within the scope and aims of the study, the statistical analysis has been performed appropriately, but its rigor is limited by design and should be interpreted as descriptive rather than evaluative. The authors correctly use descriptive statistics (frequencies, percentages, means and standard deviations) to summarize feasibility, coverage, and satisfaction outcomes in a cross-sectional feasibility study, and no inappropriate inferential claims are made. However, the analysis remains basic, with no comparative or inferential testing, no assessment of associations, and no validation of measurement scales, which limits the ability to draw conclusions beyond descriptive feasibility and acceptability and should be explicitly reflected in the framing of results and conclusions.

Thank you for this comment. We agree that the analysis should be interpreted as descriptive rather than evaluative. We have now clarified that the analysis was descriptive, that Likert-scale items were analyzed individually, and that the study was not intended to evaluate effectiveness or causal impact (page 7, lines 114–123; page 9, lines 158–168; page 20, lines 383–388).

56. Overall, the manuscript is clearly structured, logically organized, and generally written in understandable standard English, and the main messages are easy to follow. The flow from introduction through methods, results, and discussion is coherent, and tables and figures are appropriately used to support the text. However, the manuscript would benefit from careful language editing to address minor grammatical errors, awkward phrasing, repetition, and occasional inconsistencies in tense and word choice, particularly in the Introduction and Discussion sections. These issues do not obscure the scientific content but should be corrected at revision to improve clarity, precision, and readability in line with PLOS ONE requirements.

Thank you. We have revised the manuscript to improve clarity, precision, grammar, and consistency in tense and wording.

**Reviewer #8**

Abstract
57. Abstract language is that of operational report not scientific (eg 'high satisfaction', 'improved tracking')
Thank you. We have revised the Abstract to use more neutral scientific language and avoid operational-report style wording.

58. The conclusion "demonstrates feasibility, acceptability, and scalability" cannot be made from your study because 1) scalability was not studied, 2) no cost analysis, 3) no infrastructure assessment, 4) no health system readiness evaluation.

Thank you for this observation. We agree and have now revised the manuscript to state that further studies are needed to assess implementation challenges, effectiveness, and scalability across diverse settings (page 3, lines 55–58; page 20, lines 383–388).

Methodology
59. Proxy indicator for feasibility and scalability (i.e prevalence of caregivers lacking vaccination records (16%)) may be weak conceptually.

Thank you for this comment. We acknowledge this concern. We have revised the Methods to clarify that the initial estimate was used only to guide recruitment and that the study was descriptive and not intended for formal inference about feasibility, effectiveness, or scalability (page 7–8, lines 124–142).

60. Study is heavily centred on caregiver and provider satisfaction, 5-point Likert scale where 0 represented “not at all satisfied” and 5 represented “highly satisfied” That is actually a 6-point scale (0–5). This inconsistency portends measurement imprecision

Thank you for identifying this error. The Likert scale used was a 5-point scale ranging from 1 to 5. The scale is now consistently described as a 5-point scale ranging from 1 to 5, with clear response anchors (page 8, lines 151–157; page 12, lines 227–230; page 14, lines 249–252).

61. The study lack details of qualitative data analysis

Thank you. We have now clarified that open-ended responses were not analyzed as formal qualitative data but were reviewed and descriptively summarized to identify commonly reported issues and experiences (page 9, lines 166–168, page 17, lines 326-328).

62. Ethics section incomplete - ethics reference number missing no mention of data confidentiality procedures

Thank you. We have now added the ethics approval reference number and data confidentiality procedures ((page 9, lines 170–175).

63. If purposive sampling was used as indicated in the discussion: Sample size calculation using Z²pq/d² may be invalid, representativeness is not statistically justified

Thank you for raising this point. We agree that purposive sampling limits statistical representativeness. We have revised the Methods to clarify that the estimate guided recruitment only, that purposive sampling was used to include participants with direct experience of the RCC EIR, and that the study was not designed for statistical inference (page 7–9, lines 124–168).

In addition, we have removed the explicit sample size formula from the manuscript, as its inclusion could be misinterpreted as supporting statistical inference, whereas this study was designed as a descriptive assessment using purposive sampling.

**Reviewer #9**

64. This study is not methodologically corrected. Lack of proper sample size estimation, clarification of sample collection process and faulty analysis are present, which is a serious concern. I recommended a rejection for this manuscript. See the attachment for details.

Thank you for this detailed and critical assessment. We appreciate the concerns raised regarding sample size estimation, sampling strategy, and analytical approach. We have revised the manuscript substantially to improve methodological clarity and transparency. We have now:

- clarified that the study was designed as a descriptive, cross-sectional assessment of perceived feasibility and acceptability, rather than an inferential or effectiveness study
- revised the sampling approach, emphasizing the use of purposive sampling to include participants with direct experience of the EIR
- removed elements that could imply statistical inference, including explicit emphasis on sample size calculation parameters
- expanded the data collection description, including questionnaire design and pretesting
- clarified the descriptive analytical approach and aligned the Results and Discussion accordingly
- strengthened the limitations section to explicitly acknowledge sampling bias and limited generalizability

We believe these revisions improve internal consistency and ensure that the study design, analysis, and interpretation are now properly framed (page 7–9, lines 114–175; page 15–20, lines 277–388).

Materials and Methods

65. As there is no previous study, it is recommended to calculate as 50%. But this study calculates 16% which is based on caregivers who are not vaccinated. If I suggest to calculate the sample size again with 50% as prevalence rate.

Thank you for this comment. We have now revised the Methods to clarify that the estimate was used to guide recruitment only and was not intended to support formal statistical inference. We also clarified that purposive sampling was used because the study required participants with direct experience of the RCC EIR (page 7–8, lines 124–142).

66. Data analysis section is lack of proper rigorous analysis.

Thank you. We acknowledge that the analysis is limited to descriptive statistics and does not include inferential or comparative analyses. This was because the study was designed as an exploratory, cross-sectional assessment of perceived feasibility and acceptability, rather than to evaluate effectiveness or test associations. We have revised the Methods section to clarify the analytical approach and ensured that the Results and Discussion avoid overinterpretation and are consistent with the descriptive nature of the study (page 9, lines 158–168).

67. Ethical approval was taken but there is no approval ID.

Thank you for this observation. We have now included the ethics approval reference number and clarified data confidentiality procedures (page 9, lines 170–175).

68. Lack of description about data collection process, using tools, design questionnaire, validation of questionnaire, any pilot survey before collection of data and detailed information about this entire process.

Thank you for this comment. We have now expanded the Data collection section to describe the semi-structured questionnaire, closed- and open-ended questions, pretesting, minor modifications, and separate sections for caregivers and HCPs (page 8, lines 143–157).

Results

69. I would like to suggest authors to add a demographic table for this with 95% CI

Thank you for this suggestion. We agree that confidence intervals are useful for inferential studies. However, because this study used purposive sampling and was designed as a descriptive assessment, confidence intervals could imply population-level inference that the study design does not support. We therefore retained descriptive reporting and clarified the descriptive analytical approach (page 9, lines 158–168).

70. How target achieved was calculated as sample was 207. Similarly, methodology said their sample size was 207 but they collected 321 data. There is a lack of proper validation for this.

Thank you. We have now clarified that the initial estimate of approximately 207 caregivers was used to guide recruitment, whereas the final sample included 321 participants because recruitment included eligible caregivers and HCPs available during the data collection period. We avoided presenting the sample as statistically representative (page 7–8, lines 124–142).

71. Table 2 mentioned 5-level likert but there is missing other two.

Thank you for this comment. Categories with zero responses were not included in the original tables for brevity. We have now revised Tables 2 and 3 to include all five Likert response categories, including categories with zero responses (page 12, lines 226–230; page 13–14, lines 247–252).

**Reviewer 10**

72. It is mentioned that the EIR was piloted in 2019, but the study was conducted in 2024 which results in a 5 years gap. The manuscript did not clarify whether the assessment of feasibility and acceptability covers the whole implementation period or only recent performance.

Thank you. We have now clarified that the assessment reflects user perceptions and reported operational experiences during the 2024 data collection period rather than the full implementation period since 2019 (page 7, lines 118–120).

73. In line 125, 100% vaccination coverage was mentioned which seems implausibly high. It might be better to explain sample representativeness or verification methods.

Thank you for raising this concern. We have now clarified that the figures apply only to children registered in the EIR study sample and should not be interpreted as population-level coverage estimates (page 10, lines 183–190).

74. Satisfaction was entirely based on self-reported data. It would be better to have objective verification such as vaccination card checks, registry audits or some kind of observation of the system use.

Thank you for this comment. We agree that satisfaction findings were based on self-reported data and may be affected by recall or social desirability bias. We have acknowledged this limitation in the Discussion and noted that future studies should include objective data sources, such as registry audits (page 17, lines 319–328).

75. The manuscript did not mention any inter-rater reliability or data quality checks.

Thank you for this comment. As the study used descriptive analysis and open-ended responses were summarized descriptively, we did not conduct formal inter-rater reliability testing. We have clarified the descriptive nature of this component and acknowledged the need for richer qualitative exploration in future studies (page 9, lines 166–168; page 17, lines 326–328)

76. The statistical analysis only included descriptive statistics. It could benefit from bivariate analysis, comparing outcomes across different groups such as registered in the venue vs home visits. Only descriptive statistics are presented. The study would benefit from:

Thank you for this suggestion. Given the descriptive and exploratory nature of the study and the small HCP sample, we did not conduct inferential or bivariate analyses. We have now clarified this in the Methods (page 9, lines 158–168).

77. HCPs reported several technical and operational challenges during the EIR submission process, with the most common being slow internet connectivity (31%), and combined slow internet with tablet issues (37%) (Figure 3, Panel A). Tablet malfunctions were independently reported by 19%"---> the manuscript doesn't discuss anything about mitigation strategies, system downtime frequency, or workarounds used by providers.

Thank you. We have expanded the Discussion to include mitigation strategies. However, system downtime frequency and specific workarounds were not systematically collected, and we have avoided adding unsupported details (page 19, lines 352–365).

78. The manuscript did not mention any description of the EIR system architecture, features, or data flow.

Thank you for this helpful suggestion. We have now added a brief description of EIR features and workflow, including unique identifiers, real-time data entry using tablets, and automated SMS reminders. We also clarified caregiver and HCP roles (page 7, lines 120–123).

79. The discussion or result section did not mention details on how qualitative data were analyzed beyond "descriptive summary".

Thank you. We have now clarified that open-ended responses were reviewed and descriptively summarized to identify commonly reported issues and experiences (page 9, lines 166–168).

80. The manuscript does not mention anything about data privacy, security measures for the electronic registry, or how personally identifiable information is protected.

Thank you. We have now added information on anonymization and exclusion of personally identifiable information from the study dataset (page 9, lines 171–175).

**Reviewer #11**

81. This is an interesting manuscript exploring the implementation of EIR to improve immunization coverage and monitoring in Bangladesh, as stated in the submitted manuscript. Overall, the research question is addressed using appropriate methods, and the results are properly presented. Nevertheless, a description of the current workflow and data flow of the immunization program in the studied health facilities would support better understanding of the context and the barriers and advantages presented. The reader cannot assess the current integration of the digital system if this information is not provided.

Thank you for this helpful comment. We agree and have now added a brief description of EIR features and workflow in the Methods, including unique identifiers, real-time tablet-based data entry, automated SMS reminders, and the distinction between caregivers as recipients of EIR-supported services and HCPs as direct users of the digital platform (page 7, lines 120–123).

Abstract:
82. Introduction: “We assessed the feasibility of the EIR to provide evidence and inform policymakers on strategies for scaling up digital immunization systems in Bangladesh.” I find this statement inaccurate and it does not represent the main focus of the paper, as informing policy makers was not part of the results of the paper. I recommend revising and focusing on the main focus of the research in the abstract methods section.

Thank you for this comment. We have revised the Abstract to remove statements implying policy influence or national scale-up as a study objective. The Abstract now focuses on perceptions related to the feasibility and acceptability of EIR-supported services within the urban pilot setting (page 2, lines 20–30).

83. “Coverage Evaluation Surveys 2023 revealed gaps 60 between crude and valid vaccination coverage, …” : specify the differences between the two and the specific gaps that were identified and can be supported by electronic immunization registries. This paragraph overall needs some revision to guide the reader to the specific evidence on the current gaps and the barriers and which of those can specifically be solved by scaling up electronic immunization registries, as I find the current description quite vague and general, and possibly misaligned with scope of the study.

Thank you for this comment. We agree and have now revised the Introduction to clarify that gaps between crude and valid coverage may reflect delayed vaccination, missed doses, and record-keeping limitations, and that EIRs may support individual-level tracking, data availability, and reminder systems (page 5, lines 74–88).

84. “Despite growing interest in scaling up EIRs nationwide, empirical evidence on their outcomes in Bangladesh remains limited.»: it is hard to understand this statement as there is no reference. Please revise and describe exactly what is that is already evident and what is still a gap of the current implementation. It is also hard to connect the provided information of the implementation with the feasibility that is being studies.

Thank you. We agree that the original statement did not clearly distinguish what is currently known about EIR implementation in Bangladesh and what evidence gaps remain. We have now revised the Introduction to distinguish existing programmatic descriptions of EIR implementation from the remaining gap in empirical evidence on user-level perceptions and operational experiences (page 6, lines 96–107).

Introduction:
85. “Bangladesh has made remarkable progress in immunization since the launch of its Expanded Program on Immunization (EPI) in 1979, achieving impressive reductions in vaccine-preventable diseases”: it would be more accurate to provide specific numbers to support these statements on progress.

Thank you for this helpful suggestion. We have revised the Introduction to provide specific contextual information supporting the progress of Bangladesh’s immunization program. The revised text now states that coverage of key childhood vaccines has reached around 90%, alongside marked reductions in vaccine-preventable diseases (page 5, lines 74–77).

Methods
86. Data collection: the difference between the tool used for the caregivers and the one used for the care providers is not easy to understand with the description provided.

Thank you for this comment. We have revised the Methods section to clarify that the questionnaire included separate sections tailored to each group, reflecting their different roles in the EIR-supported service (page 8, lines 143–157).

87. Given the aim of the study it is difficult to understand why the study is mostly focused on caregivers (n=305 caregivers) instead of the main users of the immunization registry who are the health care providers (n=16). Also, why the data captured for the different cadres of health providers are not presented separately, given their clearly different interaction with the digital system.

Thank you for this comment. We agree that healthcare providers are the primary direct users of the EIR system, whereas caregivers are recipients of EIR-supported immunization services. Caregivers were included to assess perceptions related to service delivery components influenced by the EIR, such as registration processes, communication, reminders, and follow-up services. The larger caregiver sample reflects the service delivery context, where caregivers constitute the main recipients of routine immunization services. We have also clarified that the number of HCPs included in the study was small (n = 16), which limited the depth of provider-level analysis. Given the descriptive nature of the study and the small sample size across HCP cadres, stratified analyses by provider type were not undertaken, as such comparisons would have limited interpretability. These points have now been clarified in the Methods, Results and Discussion sections (page 7, lines 120–123; page 8, lines 148-157; page 13, lines 231–242; page 17, lines 321–322).

Results:
88. What do the following components mean for the caregivers: “easy of using the EIR system”; “contribution of EIR to the EPI program”; “suitability of EIR for nationwide implementation”. Given that caregivers are not users of the digital registry, but receivers of a service supported by the digital registry. What is the relevance of these component for the caregivers.

Thank you. We agree that caregivers were not direct users of the EIR platform and therefore interpreted these items from the perspective of their experience with EIR-supported immunization services rather than interaction with the digital registry itself. In this context, items such as “ease of using the EIR system” reflected the perceived ease of accessing EIR-supported services; “contribution of the EIR to the EPI program” reflected caregivers’ perceptions of service organization and communication; and “suitability of the EIR for nationwide implementation” reflected their views on the broader usefulness and acceptability of these services.

We have clarified this interpretation in the Results and Discussion sections by explicitly stating that caregiver responses reflected perceptions of EIR-supported service delivery rather than direct evaluation of the digital registry system (page 11, lines 207–226; page 16, lines 290–295; page 17, lines 324–326).

Discussion
89. «Vaccination coverage remained high, and all eligible children were successfully registered.”: remained high compared to what. What did the authors expect to find?

Thank you. We have revised the wording to remove any implied temporal comparison and to present the findings descriptively. We have now clarified that reported vaccination uptake among children registered in the EIR system was high within the study sample (page 10, lines 183–190).

90. “…showed strong potential for broader scale-up.”: which results are supporting this statement? Also the barriers reported are not negligible.

Thank you. We have now removed scale-up claims and revised the Conclusion to state that further studies are needed to assess implementation challenges, effectiveness, and scalability across diverse settings (page 20, lines 383–388).

91. “The high caregiver satisfaction (91%) observed in our study is consistent with evidence…”: this is not reported in the results section. Also the authors have not assessed overall satisfaction.

Thank you. We have removed the overall satisfaction statement and now report Likert responses by individual item without calculating a composite or overall satisfaction score (page 9, lines 164–165; page 11–14, lines 207–252)

92. Overall, I can observe an overinterpretation of the findings and related implications for the health system.

Thank you. We agree and have revised the manuscript throughout to avoid overinterpretation. We have reframed the findings as descriptive and focused on perceived feasibility and acceptability rather than system-level impact (page 7, lines 114–123; page 15–16, lines 277–295; page 20, lines 383–388).

**Reviewer #12**

93. This study is very important, as it seeks to provide evidence on the feasibility, acceptability, challenges and advantages of an EIR in Bangladesh. This can provide evidence for a national scale-up, and other LMICs can also benefit from this experience in Bangladesh. That said, I believe the manuscript can benefit from some revisions in the focus and analysis of the results to make it more robust.

Thank you for your encouraging comment. We have revised the manuscript to improve focus, clarify the descriptive analysis, and ensure that the Discussion and Conclusion are aligned with the results.

94. Consider revising your topic to reflect the content of the study. Most of the data reported in the manuscript speaks to caregiver and HCP satisfaction, not the feasibility of the EIR.

Thank you for this comment. We retained the title but revised the framing throughout the manuscript to clarify that the study assesses perceptions related to feasibility and acceptability, with satisfaction interpreted as one component of acceptability rather than the sole outcome (page 2, lines 27–30; page 9, lines 151–157; page 15–16, lines 277–295).

95. I recommend that the authors consider reporting this study in line with at least one of the WHO digital health evaluation frameworks. This will make it easier to compare with other studies in different settings in the LMICs.

We appreciate this suggestion. Because the study was already completed and the available data were not collected prospectively using a digital health evaluation framework, we did not retrospectively impose one. Instead, we strengthened the descriptive framing and clarified the limitations of the evaluation.

96. I recommend that the authors clarify whether they are measuring the feasibility or satisfaction or both. Such clarity would help to focus on the substance of the study. Starting from the title of the study, through the results and discussions, ensure consistency.

Thank you for this comment. We have now clarified that the study assessed perceptions related to feasibility and acceptability of EIR-supported services, and that satisfaction was interpreted as one component of acceptability (page 8, lines 151–157).

97. Report the Cronbach's alpha (internal consistency) of the Likert scale here (Lines 101-103).

Thank you for this comment. We agree that internal consistency measures such as Cronbach’s alpha are appropriate for validated scales designed to measure a single underlying construct. However, in this study, the Likert items were used to capture perceptions across distinct aspects of EIR-supported services (e.g., registration processes, communication, and operational experience), rather than to form a unified psychometric scale. As such, Cronbach’s alpha was not considered appropriate. We have clarified in the Methods that the analysis was descriptive and that Likert items were interpreted individually rather than combined into a composite measure (page 9, lines 164–165).

98. Conduct some advanced analytical statistics to compare the satisfaction between caregivers and HCPs (Line 106).

Thank you. Because caregivers and HCPs interacted with the EIR differently and responded to role-specific items, we did not conduct direct statistical comparisons. We have clarified this distinction in the revised manuscript (page 9, lines 158–168).

99. In line 107, you stated that you computed means and standard deviations. However, there are no means and SDs reported in your results. Include them for the satisfaction of caregivers and HCPs, respectively.

Thank you for highlighting this inconsistency. We have now revised the Data analysis section to remove unnecessary reference to means and standard deviations for Likert satisfaction findings and aligned the analysis with the results, which report frequencies and percentages (page 9, lines 159–168).

100. Insert the Ethics approval number here (111-112).

Thank you. We have now added the ethics approval reference number (page 9, lines 170–175).

101. I would recommend that the authors conduct a more advanced analysis of the data, comparing the HCP satisfaction and Caregiver satisfaction. Use an " Independent t-test (caregivers vs providers)" or any other advanced statistical method.

Thank you for this suggestion. We did not conduct this comparison because caregiver and HCP items were role-specific and not directly comparable, and because the HCP sample was small. The study was descriptive rather than inferential (page 9, lines 158–168; page 13, lines 231–242)

102. After all these revisions, the discussion and conclusion have to be adapted to the results.

Thank you. We have now revised the Discussion and Conclusion to align with the descriptive results and avoid overinterpretation (page 15–20, lines 275–388).

**Reviewer #13**

103. The paper is an interesting addition to the growing body of work on EIRs, but I suggest significant revisions before publication:

Thank you for your constructive assessment. We have revised the manuscript substantially to improve framing, methodology, interpretation, and clarity.

104. The paper and findings should be framed specifically as around perceptions of EIR feasibility and acceptability to caregivers and health care workers as there is no comparative data presented to show that EIR implementation in the pilot district has improved timeliness, coverage or other metrics of EPI effectiveness.

We agree. We have now revised the manuscript to frame the findings as perceptions and operational experiences related to feasibility and acceptability, not evidence of improved timeliness, coverage, or EPI effectiveness. We also have added a clear statement that the study was not designed to evaluate effectiveness or causal impact (page 7, lines 114–123; page 15–16, lines 277–295; page 20, lines 383–388).

105. unclear why sampling size is relevant as only descriptive analysis with no comparison presented

Thank you for this comment. We have now clarified that the initial sample size estimate was used only to guide recruitment and not to support hypothesis testing or statistical inference (page 7–8, lines 124–142).

106. I would expect EIR feasibility and acceptance among HCWs, rather than caregivers, as HCWs are the ones primarily using the EIR. Yet number of HCWs in the sample is very small, also making it hard to draw valid conclusions on a quantitative basis

Thank you for this comment. We agree and have now clarified that healthcare providers were the direct system users, while caregivers were recipients of EIR-supported services. We also explicitly acknowledged the small healthcare provider sample as a limitation (page 7, lines 120–123; page 13, lines 231–242; page 17, lines 321–322).

107. Please present all data collected for each Likert category, even if number is 0 - e.g., some tables only 3/5 likert categories are included

Thank you. Tables 2 and 3 have now been revised to present all five Likert response categories, including categories with zero responses (page 12, lines 226–230; page 13–14, lines 247–252).

108. consider if (i) qualitative data to enhance understanding of HCW perceptions can be collected; and / or (ii) data from other regions or time periods can be used to validate / assess metrics such as timeliness more objectively.

We agree and have now added this as a future research recommendation, including longitudinal or comparative designs, objective registry audits, richer qualitative exploration of HCP experiences, and assessment across diverse geographic settings (page 17, lines 326–328).

109. Remove statements such as "all eligible children were successfully registered" if it cannot be clearly described how this was verified with data outside of the EIR.

Thank you for this comment. We have removed wording implying complete registration or population-level coverage and revised the text to explicitly state that coverage findings apply only to children registered in the EIR study sample (page 10, lines 183–190; page 16, lines 290–295).

We would like to thank the reviewers and editorial team for their considered comments, which have helped us improve our paper. The revised manuscript has been reviewed and approved by all the co-authors. We believe we have responded to all the points made and hope the manuscript now meets with your approval.

Please feel free to contact me if you require any further clarification.

Yours sincerely,

Dr Nazim Uzzaman, MBBS, MPH, PhD

Adjunct Faculty, Rajshahi Medical University

Rajshahi, Bangladesh

Email: [nazim0700@gmail.com](mailto:nazim0700@gmail.com)
