# Supplementary material for: Feasibility and acceptability of an electronic immunization registry in urban Bangladesh
Source: PLoS One. 2026 Jul 2;21(7):e0352972. doi: 10.1371/journal.pone.0352972 (PMC13327150; doi:10.1371/journal.pone.0352972)
Supplement: Peer review history

# Peer review: Decision Letter 0

**DOI:** 10.1371/journal.pone.0352972.r001

**Authors:** Samuel Kofi Tchum

16 Feb 2026

PONE-D-25-67616

Feasibility and acceptability of an electronic immunization registry in urban Bangladesh

PLOS One

Dear Dr. Nazim Uzzaman,

Thank you for submitting your manuscript to PLOS ONE. After careful consideration, we feel that it has merit but does not fully meet PLOS ONE’s publication criteria as it currently stands. Therefore, we invite you to submit a revised version of the manuscript that addresses the points raised during the review process.

**Reviewer 1**

Major Comments

Generalizability

The study is limited to a single urban pilot site (Rajshahi City Corporation). Findings may not reflect rural or resource-constrained areas. Please expand the discussion on how infrastructural differences (e.g., internet connectivity, staffing) might affect nationwide rollout.

Study Design

The cross-sectional design provides only a snapshot in time. Longitudinal or comparative studies would be needed to assess sustainability, dropout reduction, and equity impacts. Please acknowledge this limitation more explicitly.

Sampling Bias

Purposive sampling may have introduced bias, particularly if participants were more likely to be favorable toward the EIR. Clarification on participant selection criteria would strengthen the methodology.

Self-reported Satisfaction

Satisfaction measures rely on self-report, which may be subject to recall and social desirability bias. Please discuss this limitation in more depth and consider triangulating with objective usage data in future studies.

Absence of Control Group

Without comparison to paper-based registries, it is difficult to attribute improvements solely to the EIR. Acknowledging this limitation would improve transparency.

Technical Challenges

While slow internet and device malfunctions are noted, the discussion could be expanded to include potential solutions (e.g., offline-ready systems, infrastructure investment, refresher training). This would strengthen the scalability argument.

Data Availability

The statement “all relevant data are within the manuscript” may limit reproducibility. Consider depositing anonymized datasets in a public repository to align with open science practices.

Minor Comments

Clarity of Writing

Some sections of the discussion repeat global evidence without fully connecting back to the Bangladesh context. Strengthening the local interpretation would enhance impact.

Figures and Tables

Figures summarizing caregiver and HCP satisfaction could benefit from clearer legends and consistent formatting of percentages.

Methodological Detail

Please provide more information on questionnaire validation and how qualitative responses were coded.

Terminology Consistency

Ensure consistent use of abbreviations (e.g., EIR, EPI) throughout the manuscript.

Ethical and Publication Considerations

Ethical approval and informed consent are clearly stated; no concerns identified.

No evidence of dual publication or ethical violations.

Financial disclosure and competing interests are appropriately declared.

Overall Recommendation

This manuscript makes a meaningful contribution to the literature on digital health and immunization in LMICs. With revisions to strengthen methodological transparency, expand discussion of limitations, and improve data accessibility, the paper will be well-positioned for publication.

**Reviewer 2**

This manuscript presents a cross-sectional assessment of the feasibility and acceptability of an electronic immunization registry (EIR) in urban Bangladesh. The topic is relevant, the study appears technically sound, and the conclusions are generally supported by the data. However, several points require clarification.

Major points:

Funding statement: There is an inconsistency between the Funding and Acknowledgements sections. The authors should clarify whether WHO-IVD support was financial or in-kind and ensure consistency between these sections.

Data availability: For a survey-based study, PLOS data policy typically requires availability of the underlying de-identified dataset. The authors should clarify whether the raw survey data can be shared and update the Data Availability statement accordingly.

Minor points:

The description of the Likert scale is inconsistent (described as 5-point but defined from 0 to 5) and should be corrected.

Satisfaction outcomes are reported without explicit dissatisfied categories; the authors should clarify how these were handled and how overall satisfaction was calculated.

Minor typographical and formatting issues should be addressed.

**Reviewer 3**

Study design and causal interpretation

The cross-sectional design of the study limits the ability to attribute the observed high vaccination coverage and improved registration timeliness directly to the electronic immunization registry. Several statements in the Abstract, Results, and Conclusions sections appear to imply an impact of the EIR rather than focusing strictly on feasibility and acceptability. The conclusions should be reframed to clearly distinguish association from causation and to avoid over-claiming system effectiveness.

Sampling strategy and potential bias

Caregivers were selected using purposive sampling, and all participants were already engaged with the EIR system. This approach introduces selection bias and likely inflates estimates of satisfaction and vaccination coverage. The limitations section should be expanded to explicitly discuss how purposive sampling and the exclusion of non-users may have influenced the findings.

Vaccination coverage results (100% coverage)

The reporting of 100% vaccination coverage for multiple antigens in an urban setting is unusual and requires careful contextualization. It is unclear whether the reported coverage reflects true population-level coverage or only coverage among children registered in the EIR system. The authors should clarify the denominators used, explicitly state that coverage estimates apply only to registered children, and temper interpretations accordingly.

Very small healthcare provider sample size

Only 16 healthcare providers were included in the study, yet results are presented in percentages and used to support relatively strong conclusions. This small sample size limits both statistical reliability and interpretive robustness. The manuscript should more explicitly acknowledge this limitation and avoid broad generalizations based on these data.

Data availability statement

The manuscript states that all relevant data are contained within the manuscript, yet the results appear to be derived from underlying survey datasets. This may not fully align with PLOS ONE data transparency requirements. The authors should clarify whether anonymized datasets can be shared as supplementary files or deposited in a public repository.

Minor reviewer comments (technical and editorial)

Likert scale description

The Likert scale is described in the Methods section as ranging from 0 to 5, while the tables appear to reflect a 1 to 5 scale. This discrepancy should be clarified and standardized throughout the manuscript.

Figures and tables

There are inconsistencies in figure numbering in the text, such as references to Figure 1 and Figure 3. These should be checked and corrected. In addition, figure captions should be improved to be fully self-explanatory without requiring reference to the main text.

**Reviewer 4**

Overall Assessment

This manuscript presents a cross-sectional evaluation of the feasibility and acceptability of an Electronic Immunization Registry (EIR) piloted in an urban setting in Bangladesh. The topic is timely and relevant, particularly in the context of digital health scale-up in low- and middle-income countries (LMICs) aligned with Immunization Agenda 2030. The study contributes descriptive evidence on user satisfaction, registration timeliness, and operational challenges from both caregivers and healthcare providers. While the findings are generally positive and policy-relevant, limitations in study design, sampling strategy, and inferential depth constrain the strength of conclusions regarding effectiveness and scalability. With substantive revisions to clarify methodology, contextualize findings, and temper conclusions, the manuscript could be suitable for publication.

Major Comments

Study design limits causal inference and claims of improvement

The cross-sectional design without a comparator (e.g., pre-EIR baseline or paper-based registry group) limits the ability to attribute high vaccination coverage, registration timeliness, or satisfaction directly to the EIR. Statements suggesting that the EIR “improved” coverage or service delivery should be reframed more cautiously to reflect association rather than impact.

Sampling strategy and representativeness require clearer justification

The use of purposive sampling to recruit “information-rich” caregivers and inclusion of all HCPs involved in the EIR introduces potential selection bias. The manuscript should more explicitly discuss how this approach may overestimate satisfaction and feasibility, and how findings may differ among less-engaged users or marginalized populations.

Interpretation of vaccination coverage results may be overstated

Reported vaccination coverage rates (100% for several antigens) are exceptionally high and may reflect programmatic selection rather than population-level performance. The manuscript should clarify whether these figures represent verified coverage within the EIR cohort only, and should avoid implying general population coverage improvements.

Limited analytical depth beyond descriptive statistics

The analysis is restricted to descriptive statistics, which is appropriate for feasibility assessment but limits insight into factors associated with satisfaction, delayed registration, or reported challenges. The authors should either justify this limitation explicitly or explore simple stratified analyses (e.g., caregiver demographics vs. satisfaction) if data permit.

Minor Comments

Clarify Likert scale description and reporting

The Likert scale is described as ranging from 0 to 5, but tables appear to reflect only categorical responses without explicit mention of scale anchors. Consistency and clarity in scale description would improve transparency.

Improve clarity in tables and figures

Tables 2 and 3 would benefit from clearer denominators and explicit reporting of missing or neutral responses. Figure captions should specify whether percentages are mutually exclusive or allow multiple responses.

Recommendation

The study is relevant and methodologically sound for a feasibility assessment, but substantial revisions are needed to address overinterpretation of results, clarify sampling limitations, and align conclusions more closely with the descriptive nature of the data.

**Reviewer 5**

This is very good research for the Bangladeshi population. This type of research should be performed. If this article publishes in national journal, then the effectiveness will be more for the national population. Thank you.

**Reviewer 6**

The authors are advised to visit the journal website for proper structuring of the article. The article is recommended to be accepted for publication if updated accordingly. The review is uploaded as an attachment.

**Reviewer 7**

The manuscript presents a technically sound feasibility study, with an appropriate study design and descriptive analyses that are aligned with the stated objectives. The data supports the conclusions regarding feasibility and acceptability of the EIR among caregivers and healthcare providers, and the authors appropriately acknowledge key methodological limitations. However, the manuscript would benefit from a clearer description of how caregivers interact with the EIR system, as caregivers appear to be largely passive recipients of EIR outputs rather than active users, which should be more clearly reflected in the interpretation of caregiver-related findings.

- Within the scope and aims of the study, the statistical analysis has been performed appropriately, but its rigor is limited by design and should be interpreted as descriptive rather than evaluative. The authors correctly use descriptive statistics (frequencies, percentages, means and standard deviations) to summarize feasibility, coverage, and satisfaction outcomes in a cross-sectional feasibility study, and no inappropriate inferential claims are made. However, the analysis remains basic, with no comparative or inferential testing, no assessment of associations, and no validation of measurement scales, which limits the ability to draw conclusions beyond descriptive feasibility and acceptability and should be explicitly reflected in the framing of results and conclusions.

- Overall, the manuscript is clearly structured, logically organized, and generally written in understandable standard English, and the main messages are easy to follow. The flow from introduction through methods, results, and discussion is coherent, and tables and figures are appropriately used to support the text.

However, the manuscript would benefit from careful language editing to address minor grammatical errors, awkward phrasing, repetition, and occasional inconsistencies in tense and word choice, particularly in the Introduction and Discussion sections. These issues do not obscure the scientific content but should be corrected at revision to improve clarity, precision, and readability in line with PLOS ONE requirements.

**Reviewer 8**

Dear Authors

Thank you for the efforts

Kindly find my comments below

ABSTRACT

Abstract language is that of operational report not scientific (eg 'high satisfaction', 'improved tracking')

the conclusion "demonstrates feasibility, acceptability, and scalability" cannot be made from your study because

1. Scalability was not studied

2. No cost analysis

3. No infrastructure assessment

4. No health system readiness evaluation

Methodology

1. Proxy indicator for feasibility and scalability (i.e prevalence of caregivers lacking vaccination records (16%)) may be weak conceptually.

2. Study is heavily centred on caregiver and provider satisfaction, 5-point Likert scale where 0 represented “not at all satisfied” and 5 represented “highly satisfied” That is actually a 6-point scale (0–5). This inconsistency portends measurement imprecision

3. The study lack details of qualitative data analysis

4.Ethics section incomplete - ethics reference number missing no mention of data confidentiality procedures

5. If purposive sampling was used as indicated in the discussion: Sample size calculation using Z²pq/d² may be invalid, representativeness is not statistically justified

**Reviewer 9**

This study is not methodologically corrected. Lack of proper sample size estimation, clarification of sample collection process and faulty analysis are present, which is a serious concern. I recommended a rejection for this manuscript.

see the attachment for details

**Reviewer 10**

1. It is mentioned that the EIR was piloted in 2019, but the study was conducted in 2024 which results in a 5 years gap. The manuscript did not clarify whether the assessment of feasibility and acceptability covers the whole implementation period or only recent performance.
  2. In line 125, 100% vaccination coverage was mentioned which seems implausibly high. It might be better to explain sample representativeness or verification methods.
  3. Satisfaction was entirely based on self-reported data. It would be better to have objective verification such as vaccination card checks, registry audits or some kind of observation of the system use.
  4. The manuscript did not mention any inter-rater reliability or data quality checks.
  5. The statistical analysis only included descriptive statistics. It could benefit from bivariate analysis, comparing outcomes across different groups such as registered in the venue vs home visits.
  Only descriptive statistics are presented. The study would benefit from:
  6. "HCPs reported several technical and operational challenges during the EIR submission process, with the most common being slow internet connectivity (31%), and combined slow internet with tablet issues (37%) (Figure 3, Panel A). Tablet malfunctions were independently reported by 19%"---> the manuscript doesn't discuss anything about mitigation strategies, system downtime frequency, or workarounds used by providers.
  7. The manuscript did not mention any description of the EIR system architecture, features, or data flow.
  8. The discussion or result section did not mention details on how qualitative data were analyzed beyond "descriptive summary".
  9. The manuscript does not mention anything about data privacy, security measures for the electronic registry, or how personally identifiable information is protected.

**Reviewer 11**

This is an interesting manuscript exploring the implementation of EIR to improve immunization coverage and monitoring in Bangladesh, as stated in the submitted manuscript. Overall, the research question is addressed using appropriate methods, and the results are properly presented. Nevertheless, a description of the current workflow and data flow of the immunization program in the studied health facilities would support better understanding of the context and the barriers and advantages presented. The reader cannot assess the current integration of the digital system if this information is not provided.

Abstract:

- Introduction: “We assessed the feasibility of the EIR to provide evidence and inform policymakers on strategies for scaling up digital immunization systems in Bangladesh.” I find this statement inaccurate and it does not represent the main focus of the paper, as informing policy makers was not part of the results of the paper. I recommend revising and focusing on the main focus of the research in the abstract methods section.

- “Coverage Evaluation Surveys 2023 revealed gaps 60 between crude and valid vaccination coverage, …” : specify the differences between the two and the specific gaps that were identified and can be supported by electronic immunization registries. This paragraph overall needs some revision to guide the reader to the specific evidence on the current gaps and the barriers and which of those can specifically be solved by scaling up electronic immunization registries, as I find the current description quite vague and general, and possibly misaligned with scope of the study.

- “Despite growing interest in scaling up EIRs nationwide, empirical evidence on their outcomes in Bangladesh remains limited.»: it is hard to understand this statement as there is no reference. Please revise and describe exactly what is that is already evident and what is still a gap of the current implementation. It is also hard to connect the provided information of the implementation with the feasibility that is being studies.

Introduction:

- “Bangladesh has made remarkable progress in immunization since the launch of its Expanded Program on Immunization (EPI) in 1979, achieving impressive reductions in vaccine-preventable diseases”: it would be more accurate to provide specific numbers to support these statements on progress.

Methods

- Data collection: the difference between the tool used for the caregivers and the one used for the care providers is not easy to understand with the description provided.

- Given the aim of the study it is difficult to understand why the study is mostly focused on caregivers (n=305 caregivers) instead of the main users of the immunization registry who are the health care providers (n=16). Also, why the data captured for the different cadres of health providers are not presented separately, given their clearly different interaction with the digital system.

Results:

- What do the following components mean for the caregivers: “easy of using the EIR system”; “contribution of EIR to the EPI program”; “suitability of EIR for nationwide implementation”. Given that caregivers are not users of the digital registry, but receivers of a service supported by the digital registry. What is the relevance of these component for the caregivers.

Discussion

- «Vaccination coverage remained high, and all eligible children were successfully registered.”: remained high compared to what. What did the authors expect to find?

- “…showed strong potential for broader scale-up.”: which results are supporting this statement? Also the barriers reported are not negligible.

- “The high caregiver satisfaction (91%) observed in our study is consistent with evidence…”: this is not reported in the results section. Also the authors have not assessed overall satisfaction.

- Overall I can observe an overinterpretation of the findings and related implications for the health system.

**Reviewer 12**

General comments:

This study is very important, as it seeks to provide evidence on the feasibility, acceptability, challenges and advantages of an EIR in Bangladesh. This can provide evidence for a national scale-up, and other LMICs can also benefit from this experience in Bangladesh.

That said, I believe the manuscript can benefit from some revisions in the focus and analysis of the results to make it more robust.

Specific Comments:

1. Consider revising your topic to reflect the content of the study. Most of the data reported in the manuscript speaks to caregiver and HCP satisfaction, not the feasibility of the EIR.

2. I recommend that the authors consider reporting this study in line with at least one of the WHO digital health evaluation frameworks. This will make it easier to compare with other studies in different settings in the LMICs.

3. I recommend that the authors clarify whether they are measuring the feasibility or satisfaction or both. Such clarity would help to focus on the substance of the study. Starting from the title of the study, through the results and discussions, ensure consistency.

4. Report the Cronbach's alpha (internal consistency) of the Likert scale here (Lines 101-103).

5. Conduct some advanced analytical statistics to compare the satisfaction between caregivers and HCPs (Line 106).

6. In line 107, you stated that you computed means and standard deviations. However, there are no means and SDs reported in your results. Include them for the satisfaction of caregivers and HCPs, respectively.

7. Insert the Ethics approval number here (111-112).

8. I would recommend that the authors conduct a more advanced analysis of the data, comparing the HCP satisfaction and Caregiver satisfaction. Use an " Independent t-test (caregivers vs providers)" or any other advanced statistical method.

9. After all these revisions, the discussion and conclusion have to be adapted to the results.

**Reviewer 13**

The paper is an interesting addition to the growing body of work on EIRs, but I suggest significant revisions before publication:

- the paper and findings should be framed specifically as around perceptions of EIR feasibility and acceptability to caregivers and health care workers as there is no comparative data presented to show that EIR implementation in the pilot district has improved timeliness, coverage or other metrics of EPI effectiveness.

- unclear why sampling size is relevant as only descriptive analysis with no comparison presented

- I would expect EIR feasibility and acceptance among HCWs, rather than caregivers, as HCWs are the ones primarily using the EIR. Yet number of HCWs in the sample is very small, also making it hard to draw valid conclusions on a quantitative basis

- Please present all data collected for each Likert category, even if number is 0 - e.g., some tables only 3/5 likert categories are included

- consider if (i) qualitative data to enhance understanding of HCW perceptions can be collected ; and / or (ii) data from other regions or time periods can be used to validate / assess metrics such as timeliness more objectively.

- Remove statements such as "all eligible children were successfully registered" if it cannot be clearly described how this was verified with data outside of the EIR.

Please submit your revised manuscript by Apr 02 2026 11:59PM. If you will need more time than this to complete your revisions, please reply to this message or contact the journal office at plosone@plos.org. When you're ready to submit your revision, log on to https://www.editorialmanager.com/pone/ and select the 'Submissions Needing Revision' folder to locate your manuscript file.

Please include the following items when submitting your revised manuscript:

- A letter that responds to each point raised by the academic editor and reviewer(s). You should upload this letter as a separate file labeled 'Response to Reviewers'.
- A marked-up copy of your manuscript that highlights changes made to the original version. You should upload this as a separate file labeled 'Revised Manuscript with Track Changes'.
- An unmarked version of your revised paper without tracked changes. You should upload this as a separate file labeled 'Manuscript'.

If you would like to make changes to your financial disclosure, please include your updated statement in your cover letter. Guidelines for resubmitting your figure files are available below the reviewer comments at the end of this letter.

If applicable, we recommend that you deposit your laboratory protocols in protocols.io to enhance the reproducibility of your results. Protocols.io assigns your protocol its own identifier (DOI) so that it can be cited independently in the future. For instructions see: https://journals.plos.org/plosone/s/submission-guidelines#loc-laboratory-protocols. Additionally, PLOS ONE offers an option for publishing peer-reviewed Lab Protocol articles, which describe protocols hosted on protocols.io. Read more information on sharing protocols at https://plos.org/protocols?utm_medium=editorial-email&utm_source=authorletters&utm_campaign=protocols.

We look forward to receiving your revised manuscript.

Kind regards,

Samuel Kofi Tchum, Ph.D.

Academic Editor

PLOS One

Journal Requirements:

When submitting your revision, we need you to address these additional requirements.

1. Please ensure that your manuscript meets PLOS ONE's style requirements, including those for file naming. The PLOS ONE style templates can be found at

https://journals.plos.org/plosone/s/file?id=wjVg/PLOSOne_formatting_sample_main_body.pdf and

https://journals.plos.org/plosone/s/file?id=ba62/PLOSOne_formatting_sample_title_authors_affiliations.pdf

2. Thank you for stating the following financial disclosure:

“The author(s) received no specific funding for this work."

At this time, please address the following queries:

a) Please clarify the sources of funding (financial or material support) for your study. List the grants or organizations that supported your study, including funding received from your institution.

b) State what role the funders took in the study. If the funders had no role in your study, please state: “The funders had no role in study design, data collection and analysis, decision to publish, or preparation of the manuscript.”

c) If any authors received a salary from any of your funders, please state which authors and which funders.

d) If you did not receive any funding for this study, please state: “The authors received no specific funding for this work.”

Please include your amended statements within your cover letter; we will change the online submission form on your behalf.

3. Thank you for stating the following in the Acknowledgments Section of your manuscript:

“We are grateful to WHO-IVD, Bangladesh for their funding support to carry out the evaluation work. We sincerely thank the study participants and the dedicated RCC and WHO staff for their invaluable support throughout this study.”

We note that you have provided funding information that is not currently declared in your Funding Statement. However, funding information should not appear in the Acknowledgments section or other areas of your manuscript. We will only publish funding information present in the Funding Statement section of the online submission form.

Please remove any funding-related text from the manuscript and let us know how you would like to update your Funding Statement. Currently, your Funding Statement reads as follows:

“The author(s) received no specific funding for this work."

Please include your amended statements within your cover letter; we will change the online submission form on your behalf.

4. Please upload a copy of Figure 3, to which you refer in your text on page 10. If the figure is no longer to be included as part of the submission please remove all reference to it within the text.

5. If the reviewer comments include a recommendation to cite specific previously published works, please review and evaluate these publications to determine whether they are relevant and should be cited. There is no requirement to cite these works unless the editor has indicated otherwise.

[Note: HTML markup is below. Please do not edit.]

Reviewers' comments:

Reviewer's Responses to Questions

**Comments to the Author**

1. Is the manuscript technically sound, and do the data support the conclusions?

The manuscript must describe a technically sound piece of scientific research with data that supports the conclusions. Experiments must have been conducted rigorously, with appropriate controls, replication, and sample sizes. The conclusions must be drawn appropriately based on the data presented.

Reviewer #1: Yes

Reviewer #2: Yes

Reviewer #3: Partly

Reviewer #4: Yes

Reviewer #5: Yes

Reviewer #6: Yes

Reviewer #7: Yes

Reviewer #8: No

Reviewer #9: No

Reviewer #10: Yes

Reviewer #11: No

Reviewer #12: Yes

Reviewer #13: Partly

**********

2. Has the statistical analysis been performed appropriately and rigorously?

Reviewer #1: Yes

Reviewer #2: Yes

Reviewer #3: Yes

Reviewer #4: Yes

Reviewer #5: Yes

Reviewer #6: Yes

Reviewer #7: Yes

Reviewer #8: No

Reviewer #9: No

Reviewer #10: No

Reviewer #11: Yes

Reviewer #12: No

Reviewer #13: Yes

**********

3. Have the authors made all data underlying the findings in their manuscript fully available?

The PLOS Data policy requires authors to make all data underlying the findings described in their manuscript fully available without restriction, with rare exception (please refer to the Data Availability Statement in the manuscript PDF file). The data should be provided as part of the manuscript or its supporting information, or deposited to a public repository. For example, in addition to summary statistics, the data points behind means, medians and variance measures should be available. If there are restrictions on publicly sharing data—e.g. participant privacy or use of data from a third party—those must be specified.

Reviewer #1: Yes

Reviewer #2: No

Reviewer #3: Yes

Reviewer #4: Yes

Reviewer #5: Yes

Reviewer #6: Yes

Reviewer #7: Yes

Reviewer #8: Yes

Reviewer #9: No

Reviewer #10: Yes

Reviewer #11: Yes

Reviewer #12: Yes

Reviewer #13: Yes

**********

4. Is the manuscript presented in an intelligible fashion and written in standard English?

PLOS ONE does not copyedit accepted manuscripts, so the language in submitted articles must be clear, correct, and unambiguous. Any typographical or grammatical errors should be corrected at revision, so please note any specific errors here.

Reviewer #1: Yes

Reviewer #2: Yes

Reviewer #3: Yes

Reviewer #4: Yes

Reviewer #5: Yes

Reviewer #6: Yes

Reviewer #7: Yes

Reviewer #8: No

Reviewer #9: Yes

Reviewer #10: Yes

Reviewer #11: Yes

Reviewer #12: Yes

Reviewer #13: Yes

**********

5. Review Comments to the Author

Please use the space provided to explain your answers to the questions above. You may also include additional comments for the author, including concerns about dual publication, research ethics, or publication ethics. (Please upload your review as an attachment if it exceeds 20,000 characters)

Reviewer #1: Major Comments

Generalizability

The study is limited to a single urban pilot site (Rajshahi City Corporation). Findings may not reflect rural or resource-constrained areas. Please expand the discussion on how infrastructural differences (e.g., internet connectivity, staffing) might affect nationwide rollout.

Study Design

The cross-sectional design provides only a snapshot in time. Longitudinal or comparative studies would be needed to assess sustainability, dropout reduction, and equity impacts. Please acknowledge this limitation more explicitly.

Sampling Bias

Purposive sampling may have introduced bias, particularly if participants were more likely to be favorable toward the EIR. Clarification on participant selection criteria would strengthen the methodology.

Self-reported Satisfaction

Satisfaction measures rely on self-report, which may be subject to recall and social desirability bias. Please discuss this limitation in more depth and consider triangulating with objective usage data in future studies.

Absence of Control Group

Without comparison to paper-based registries, it is difficult to attribute improvements solely to the EIR. Acknowledging this limitation would improve transparency.

Technical Challenges

While slow internet and device malfunctions are noted, the discussion could be expanded to include potential solutions (e.g., offline-ready systems, infrastructure investment, refresher training). This would strengthen the scalability argument.

Data Availability

The statement “all relevant data are within the manuscript” may limit reproducibility. Consider depositing anonymized datasets in a public repository to align with open science practices.

Minor Comments

Clarity of Writing

Some sections of the discussion repeat global evidence without fully connecting back to the Bangladesh context. Strengthening the local interpretation would enhance impact.

Figures and Tables

Figures summarizing caregiver and HCP satisfaction could benefit from clearer legends and consistent formatting of percentages.

Methodological Detail

Please provide more information on questionnaire validation and how qualitative responses were coded.

Terminology Consistency

Ensure consistent use of abbreviations (e.g., EIR, EPI) throughout the manuscript.

Ethical and Publication Considerations

Ethical approval and informed consent are clearly stated; no concerns identified.

No evidence of dual publication or ethical violations.

Financial disclosure and competing interests are appropriately declared.

Overall Recommendation

This manuscript makes a meaningful contribution to the literature on digital health and immunization in LMICs. With revisions to strengthen methodological transparency, expand discussion of limitations, and improve data accessibility, the paper will be well-positioned for publication.

Reviewer #2: This manuscript presents a cross-sectional assessment of the feasibility and acceptability of an electronic immunization registry (EIR) in urban Bangladesh. The topic is relevant, the study appears technically sound, and the conclusions are generally supported by the data. However, several points require clarification.

Major points:

Funding statement: There is an inconsistency between the Funding and Acknowledgements sections. The authors should clarify whether WHO-IVD support was financial or in-kind and ensure consistency between these sections.

Data availability: For a survey-based study, PLOS data policy typically requires availability of the underlying de-identified dataset. The authors should clarify whether the raw survey data can be shared and update the Data Availability statement accordingly.

Minor points:

The description of the Likert scale is inconsistent (described as 5-point but defined from 0 to 5) and should be corrected.

Satisfaction outcomes are reported without explicit dissatisfied categories; the authors should clarify how these were handled and how overall satisfaction was calculated.

Minor typographical and formatting issues should be addressed.

Reviewer #3: Study design and causal interpretation

The cross-sectional design of the study limits the ability to attribute the observed high vaccination coverage and improved registration timeliness directly to the electronic immunization registry. Several statements in the Abstract, Results, and Conclusions sections appear to imply an impact of the EIR rather than focusing strictly on feasibility and acceptability. The conclusions should be reframed to clearly distinguish association from causation and to avoid over-claiming system effectiveness.

Sampling strategy and potential bias

Caregivers were selected using purposive sampling, and all participants were already engaged with the EIR system. This approach introduces selection bias and likely inflates estimates of satisfaction and vaccination coverage. The limitations section should be expanded to explicitly discuss how purposive sampling and the exclusion of non-users may have influenced the findings.

Vaccination coverage results (100% coverage)

The reporting of 100% vaccination coverage for multiple antigens in an urban setting is unusual and requires careful contextualization. It is unclear whether the reported coverage reflects true population-level coverage or only coverage among children registered in the EIR system. The authors should clarify the denominators used, explicitly state that coverage estimates apply only to registered children, and temper interpretations accordingly.

Very small healthcare provider sample size

Only 16 healthcare providers were included in the study, yet results are presented in percentages and used to support relatively strong conclusions. This small sample size limits both statistical reliability and interpretive robustness. The manuscript should more explicitly acknowledge this limitation and avoid broad generalizations based on these data.

Data availability statement

The manuscript states that all relevant data are contained within the manuscript, yet the results appear to be derived from underlying survey datasets. This may not fully align with PLOS ONE data transparency requirements. The authors should clarify whether anonymized datasets can be shared as supplementary files or deposited in a public repository.

Minor reviewer comments (technical and editorial)

Likert scale description

The Likert scale is described in the Methods section as ranging from 0 to 5, while the tables appear to reflect a 1 to 5 scale. This discrepancy should be clarified and standardized throughout the manuscript.

Figures and tables

There are inconsistencies in figure numbering in the text, such as references to Figure 1 and Figure 3. These should be checked and corrected. In addition, figure captions should be improved to be fully self-explanatory without requiring reference to the main text.

Reviewer #4: Overall Assessment

This manuscript presents a cross-sectional evaluation of the feasibility and acceptability of an Electronic Immunization Registry (EIR) piloted in an urban setting in Bangladesh. The topic is timely and relevant, particularly in the context of digital health scale-up in low- and middle-income countries (LMICs) aligned with Immunization Agenda 2030. The study contributes descriptive evidence on user satisfaction, registration timeliness, and operational challenges from both caregivers and healthcare providers. While the findings are generally positive and policy-relevant, limitations in study design, sampling strategy, and inferential depth constrain the strength of conclusions regarding effectiveness and scalability. With substantive revisions to clarify methodology, contextualize findings, and temper conclusions, the manuscript could be suitable for publication.

Major Comments

Study design limits causal inference and claims of improvement

The cross-sectional design without a comparator (e.g., pre-EIR baseline or paper-based registry group) limits the ability to attribute high vaccination coverage, registration timeliness, or satisfaction directly to the EIR. Statements suggesting that the EIR “improved” coverage or service delivery should be reframed more cautiously to reflect association rather than impact.

Sampling strategy and representativeness require clearer justification

The use of purposive sampling to recruit “information-rich” caregivers and inclusion of all HCPs involved in the EIR introduces potential selection bias. The manuscript should more explicitly discuss how this approach may overestimate satisfaction and feasibility, and how findings may differ among less-engaged users or marginalized populations.

Interpretation of vaccination coverage results may be overstated

Reported vaccination coverage rates (100% for several antigens) are exceptionally high and may reflect programmatic selection rather than population-level performance. The manuscript should clarify whether these figures represent verified coverage within the EIR cohort only, and should avoid implying general population coverage improvements.

Limited analytical depth beyond descriptive statistics

The analysis is restricted to descriptive statistics, which is appropriate for feasibility assessment but limits insight into factors associated with satisfaction, delayed registration, or reported challenges. The authors should either justify this limitation explicitly or explore simple stratified analyses (e.g., caregiver demographics vs. satisfaction) if data permit.

Minor Comments

Clarify Likert scale description and reporting

The Likert scale is described as ranging from 0 to 5, but tables appear to reflect only categorical responses without explicit mention of scale anchors. Consistency and clarity in scale description would improve transparency.

Improve clarity in tables and figures

Tables 2 and 3 would benefit from clearer denominators and explicit reporting of missing or neutral responses. Figure captions should specify whether percentages are mutually exclusive or allow multiple responses.

Recommendation

The study is relevant and methodologically sound for a feasibility assessment, but substantial revisions are needed to address overinterpretation of results, clarify sampling limitations, and align conclusions more closely with the descriptive nature of the data.

Reviewer #5: This is very good research for the Bangladeshi population. This type of research should be performed. If this article publishes in national journal, then the effectiveness will be more for the national population. Thank you.

Reviewer #6: The authors are advised to visit the journal website for proper structuring of the article. The article is recommended to be accepted for publication if updated accordingly. The review is uploaded as an attachment.

Reviewer #7: - The manuscript presents a technically sound feasibility study, with an appropriate study design and descriptive analyses that are aligned with the stated objectives. The data supports the conclusions regarding feasibility and acceptability of the EIR among caregivers and healthcare providers, and the authors appropriately acknowledge key methodological limitations. However, the manuscript would benefit from a clearer description of how caregivers interact with the EIR system, as caregivers appear to be largely passive recipients of EIR outputs rather than active users, which should be more clearly reflected in the interpretation of caregiver-related findings.

- Within the scope and aims of the study, the statistical analysis has been performed appropriately, but its rigor is limited by design and should be interpreted as descriptive rather than evaluative. The authors correctly use descriptive statistics (frequencies, percentages, means and standard deviations) to summarize feasibility, coverage, and satisfaction outcomes in a cross-sectional feasibility study, and no inappropriate inferential claims are made. However, the analysis remains basic, with no comparative or inferential testing, no assessment of associations, and no validation of measurement scales, which limits the ability to draw conclusions beyond descriptive feasibility and acceptability and should be explicitly reflected in the framing of results and conclusions.

- Overall, the manuscript is clearly structured, logically organized, and generally written in understandable standard English, and the main messages are easy to follow. The flow from introduction through methods, results, and discussion is coherent, and tables and figures are appropriately used to support the text.

However, the manuscript would benefit from careful language editing to address minor grammatical errors, awkward phrasing, repetition, and occasional inconsistencies in tense and word choice, particularly in the Introduction and Discussion sections. These issues do not obscure the scientific content but should be corrected at revision to improve clarity, precision, and readability in line with PLOS ONE requirements.

Reviewer #8: Dear Authors

Thank you for the efforts

Kindly find my comments below

ABSTRACT

Abstract language is that of operational report not scientific (eg 'high satisfaction', 'improved tracking')

the conclusion "demonstrates feasibility, acceptability, and scalability" cannot be made from your study because

1. Scalability was not studied

2. No cost analysis

3. No infrastructure assessment

4. No health system readiness evaluation

Methodology

1. Proxy indicator for feasibility and scalability (i.e prevalence of caregivers lacking vaccination records (16%)) may be weak conceptually.

2. Study is heavily centred on caregiver and provider satisfaction, 5-point Likert scale where 0 represented “not at all satisfied” and 5 represented “highly satisfied” That is actually a 6-point scale (0–5). This inconsistency portends measurement imprecision

3. The study lack details of qualitative data analysis

4.Ethics section incomplete - ethics reference number missing no mention of data confidentiality procedures

5. If purposive sampling was used as indicated in the discussion: Sample size calculation using Z²pq/d² may be invalid, representativeness is not statistically justified

Reviewer #9: This study is not methodologically corrected. Lack of proper sample size estimation, clarification of sample collection process and faulty analysis are present, which is a serious concern. I recommended a rejection for this manuscript.

see the attachment for details

Reviewer #10: 1. It is mentioned that the EIR was piloted in 2019, but the study was conducted in 2024 which results in a 5 years gap. The manuscript did not clarify whether the assessment of feasibility and acceptability covers the whole implementation period or only recent performance.

2. In line 125, 100% vaccination coverage was mentioned which seems implausibly high. It might be better to explain sample representativeness or verification methods.

3. Satisfaction was entirely based on self-reported data. It would be better to have objective verification such as vaccination card checks, registry audits or some kind of observation of the system use.

4. The manuscript did not mention any inter-rater reliability or data quality checks.

5. The statistical analysis only included descriptive statistics. It could benefit from bivariate analysis, comparing outcomes across different groups such as registered in the venue vs home visits.

Only descriptive statistics are presented. The study would benefit from:

6. "HCPs reported several technical and operational challenges during the EIR submission process, with the most common being slow internet connectivity (31%), and combined slow internet with tablet issues (37%) (Figure 3, Panel A). Tablet malfunctions were independently reported by 19%"---> the manuscript doesn't discuss anything about mitigation strategies, system downtime frequency, or workarounds used by providers.

7. The manuscript did not mention any description of the EIR system architecture, features, or data flow.

8. The discussion or result section did not mention details on how qualitative data were analyzed beyond "descriptive summary".

9. The manuscript does not mention anything about data privacy, security measures for the electronic registry, or how personally identifiable information is protected.

Reviewer #11: This is an interesting manuscript exploring the implementation of EIR to improve immunization coverage and monitoring in Bangladesh, as stated in the submitted manuscript. Overall, the research question is addressed using appropriate methods, and the results are properly presented. Nevertheless, a description of the current workflow and data flow of the immunization program in the studied health facilities would support better understanding of the context and the barriers and advantages presented. The reader cannot assess the current integration of the digital system if this information is not provided.

Abstract:

- Introduction: “We assessed the feasibility of the EIR to provide evidence and inform policymakers on strategies for scaling up digital immunization systems in Bangladesh.” I find this statement inaccurate and it does not represent the main focus of the paper, as informing policy makers was not part of the results of the paper. I recommend revising and focusing on the main focus of the research in the abstract methods section.

- “Coverage Evaluation Surveys 2023 revealed gaps 60 between crude and valid vaccination coverage, …” : specify the differences between the two and the specific gaps that were identified and can be supported by electronic immunization registries. This paragraph overall needs some revision to guide the reader to the specific evidence on the current gaps and the barriers and which of those can specifically be solved by scaling up electronic immunization registries, as I find the current description quite vague and general, and possibly misaligned with scope of the study.

- “Despite growing interest in scaling up EIRs nationwide, empirical evidence on their outcomes in Bangladesh remains limited.»: it is hard to understand this statement as there is no reference. Please revise and describe exactly what is that is already evident and what is still a gap of the current implementation. It is also hard to connect the provided information of the implementation with the feasibility that is being studies.

Introduction:

- “Bangladesh has made remarkable progress in immunization since the launch of its Expanded Program on Immunization (EPI) in 1979, achieving impressive reductions in vaccine-preventable diseases”: it would be more accurate to provide specific numbers to support these statements on progress.

Methods

- Data collection: the difference between the tool used for the caregivers and the one used for the care providers is not easy to understand with the description provided.

- Given the aim of the study it is difficult to understand why the study is mostly focused on caregivers (n=305 caregivers) instead of the main users of the immunization registry who are the health care providers (n=16). Also, why the data captured for the different cadres of health providers are not presented separately, given their clearly different interaction with the digital system.

Results:

- What do the following components mean for the caregivers: “easy of using the EIR system”; “contribution of EIR to the EPI program”; “suitability of EIR for nationwide implementation”. Given that caregivers are not users of the digital registry, but receivers of a service supported by the digital registry. What is the relevance of these component for the caregivers.

Discussion

- «Vaccination coverage remained high, and all eligible children were successfully registered.”: remained high compared to what. What did the authors expect to find?

- “…showed strong potential for broader scale-up.”: which results are supporting this statement? Also the barriers reported are not negligible.

- “The high caregiver satisfaction (91%) observed in our study is consistent with evidence…”: this is not reported in the results section. Also the authors have not assessed overall satisfaction.

- Overall I can observe an overinterpretation of the findings and related implications for the health system.

Reviewer #12: General comments:

This study is very important, as it seeks to provide evidence on the feasibility, acceptability, challenges and advantages of an EIR in Bangladesh. This can provide evidence for a national scale-up, and other LMICs can also benefit from this experience in Bangladesh.

That said, I believe the manuscript can benefit from some revisions in the focus and analysis of the results to make it more robust.

Specific Comments:

1. Consider revising your topic to reflect the content of the study. Most of the data reported in the manuscript speaks to caregiver and HCP satisfaction, not the feasibility of the EIR.

2. I recommend that the authors consider reporting this study in line with at least one of the WHO digital health evaluation frameworks. This will make it easier to compare with other studies in different settings in the LMICs.

3. I recommend that the authors clarify whether they are measuring the feasibility or satisfaction or both. Such clarity would help to focus on the substance of the study. Starting from the title of the study, through the results and discussions, ensure consistency.

4. Report the Cronbach's alpha (internal consistency) of the Likert scale here (Lines 101-103).

5. Conduct some advanced analytical statistics to compare the satisfaction between caregivers and HCPs (Line 106).

6. In line 107, you stated that you computed means and standard deviations. However, there are no means and SDs reported in your results. Include them for the satisfaction of caregivers and HCPs, respectively.

7. Insert the Ethics approval number here (111-112).

8. I would recommend that the authors conduct a more advanced analysis of the data, comparing the HCP satisfaction and Caregiver satisfaction. Use an " Independent t-test (caregivers vs providers)" or any other advanced statistical method.

9. After all these revisions, the discussion and conclusion have to be adapted to the results.

Reviewer #13: The paper is an interesting addition to the growing body of work on EIRs, but I suggest significant revisions before publication:

- the paper and findings should be framed specifically as around perceptions of EIR feasibility and acceptability to caregivers and health care workers as there is no comparative data presented to show that EIR implementation in the pilot district has improved timeliness, coverage or other metrics of EPI effectiveness.

- unclear why sampling size is relevant as only descriptive analysis with no comparison presented

- I would expect EIR feasibility and acceptance among HCWs, rather than caregivers, as HCWs are the ones primarily using the EIR. Yet number of HCWs in the sample is very small, also making it hard to draw valid conclusions on a quantitative basis

- Please present all data collected for each Likert category, even if number is 0 - e.g., some tables only 3/5 likert categories are included

- consider if (i) qualitative data to enhance understanding of HCW perceptions can be collected ; and / or (ii) data from other regions or time periods can be used to validate / assess metrics such as timeliness more objectively.

- Remove statements such as "all eligible children were successfully registered" if it cannot be clearly described how this was verified with data outside of the EIR.

**********

6. PLOS authors have the option to publish the peer review history of their article (what does this mean?). If published, this will include your full peer review and any attached files.

If you choose “no”, your identity will remain anonymous but your review may still be made public.

**Do you want your identity to be public for this peer review?**  For information about this choice, including consent withdrawal, please see our Privacy Policy.

Reviewer #1: **Yes:** Sura Saad Abdullah

Reviewer #2: **Yes:** Abdullah Faisal Albukhari

Reviewer #3: **Yes:** Aisha Al-Khinji

Reviewer #4: No

Reviewer #5: No

Reviewer #6: **Yes:** Surajo Adamu Wada, PhD.

Reviewer #7: No

Reviewer #8: No

Reviewer #9: No

Reviewer #10: **Yes:** Ishrat Binte Aftab

Reviewer #11: No

Reviewer #12: **Yes:** Austin Gideon Adobasom-Anane

Reviewer #13: **Yes:** Praveena Gunaratnam

**********

[NOTE: If reviewer comments were submitted as an attachment file, they will be attached to this email and accessible via the submission site. Please log into your account, locate the manuscript record, and check for the action link "View Attachments". If this link does not appear, there are no attachment files.]

To ensure your figures meet our technical requirements, please review our figure guidelines: https://journals.plos.org/plosone/s/figures

You may also use PLOS’s free figure tool, NAAS, to help you prepare publication quality figures: https://journals.plos.org/plosone/s/figures#loc-tools-for-figure-preparation.

NAAS will assess whether your figures meet our technical requirements by comparing each figure against our figure specifications.

Attachment Submitted filename: PLOS ONE_PONE-D-25-67616.docx

Attachment Submitted filename: Review-Plos One.docx

Attachment Submitted filename: Review Report_PONE-D-25-67616 .docx

Attachment Submitted filename: PONE-D-25-67616_reviewer_comments.pdf

# Author comment: Author response to Decision Letter 1

**DOI:** 10.1371/journal.pone.0352972.r002

1 Jun 2026

16th May 2026

Dr Emily Chenette

Editor-in-Chief

PLOS ONE

Re: PONE-D-25-67616R1: Feasibility and acceptability of an electronic immunization registry in urban Bangladesh

Dear Dr Chenette,

Thank you for your interest in our paper and for enclosing the encouraging and helpful comments from your reviewers. We appreciate the time and effort taken by the reviewers and the editorial team to read our manuscript and provide constructive comments to improve it. We have pleasure in submitting our revised manuscript entitled “Feasibility and acceptability of an electronic immunization registry in urban Bangladesh” to be considered for publication in PLOS ONE.

For your convenience, we have reproduced the editor’s and reviewers’ comments verbatim, before detailing our response to each of the points made. Page and line numbers refer to the ‘Revised Manuscript with Track Changes’.

Academic Editor: Dr Samuel Kofi Tchum

Thank you for submitting your manuscript to PLOS ONE. After careful consideration, we feel that it has merit but does not fully meet PLOS ONE’s publication criteria as it currently stands. Therefore, we invite you to submit a revised version of the manuscript that addresses the points raised during the review process.

Thank you for your encouraging words and for enclosing the helpful comments from your reviewers. We have responded to the reviewers’ comments in details below.

Reviewer #1

1. Generalizability: The study is limited to a single urban pilot site (Rajshahi City Corporation). Findings may not reflect rural or resource-constrained areas. Please expand the discussion on how infrastructural differences (e.g., internet connectivity, staffing) might affect nationwide rollout.

Thank you for this comment. We agree and have expanded the limitations and implementation discussion to emphasize that findings from a single urban pilot setting may not be transferable to rural or resource-constrained settings where infrastructure, staffing, and connectivity differ. We also added that nationwide implementation would require investment in internet connectivity, capacity building, and integration with broader health information systems (page 17, lines 322–328; page 19, lines 352–365; page 19, lines 375–382).

2. Study Design: The cross-sectional design provides only a snapshot in time. Longitudinal or comparative studies would be needed to assess sustainability, dropout reduction, and equity impacts. Please acknowledge this limitation more explicitly.

Thank you for this comment. We have now explicitly acknowledged this limitation. We also revised the Abstract, Methods, Discussion, and Conclusion to state that the study was not designed to evaluate effectiveness or causal impact (page 2, lines 31–40; page 7, lines 114–123; page 17, lines 313–328; page 20, lines 383–388).

3. Sampling Bias: Purposive sampling may have introduced bias, particularly if participants were more likely to be favorable toward the EIR. Clarification on participant selection criteria would strengthen the methodology.

Thank you. We agree that purposive sampling may have introduced selection bias, particularly because participants were recruited from individuals already exposed to EIR-supported services and may therefore have been more likely to report positive perceptions. We have revised the Methods section to more clearly describe the participant selection approach and rationale for using purposive sampling in this descriptive operational assessment. We have also expanded the limitations section to acknowledge that this approach may have overestimated satisfaction and perceived benefits among relatively engaged users (page 8, lines 125–142; page 17, lines 317–320).

4. Self-reported Satisfaction: Satisfaction measures rely on self-report, which may be subject to recall and social desirability bias. Please discuss this limitation in more depth and consider triangulating with objective usage data in future studies.

Thank you for this comment. We agree that self-reported satisfaction may be affected by recall and social desirability bias. We have expanded the limitations section to acknowledge this issue and have noted that future studies should include objective data sources, such as registry audits or direct observation of system use (page 17, lines 319–328).

5. Absence of Control Group: Without comparison to paper-based registries, it is difficult to attribute improvements solely to the EIR. Acknowledging this limitation would improve transparency.

Thank you for this comment. We agree that, without a comparator, observed outcomes cannot be attributed solely to the EIR. We have revised the manuscript to avoid causal language and clarify that the study provides descriptive findings (page 7, lines 114–123; page 17, lines 315–318; page 20, lines 384–388).

6. Technical Challenges: While slow internet and device malfunctions are noted, the discussion could be expanded to include potential solutions (e.g., offline-ready systems, infrastructure investment, refresher training). This would strengthen the scalability argument.

Thank you for this suggestion. We have now expanded the Discussion to include mitigation strategies, including digital infrastructure investment, offline-capable systems, and refresher training for frontline staff (page 19, lines 352–365).

7. Data Availability: The statement “all relevant data are within the manuscript” may limit reproducibility. Consider depositing anonymized datasets in a public repository to align with open science practices.

Thank you. We have now updated the Data availability statement to clarify that the de-identified dataset supporting the findings is provided as a supplementary file (page 21, lines 400–404).

8. Clarity of Writing: Some sections of the discussion repeat global evidence without fully connecting back to the Bangladesh context. Strengthening the local interpretation would enhance impact.

Thank you. We have revised the Discussion to reduce repetition of global evidence and to connect the findings to the Bangladesh implementation context, including infrastructure, workforce capacity, system integration, and phased implementation considerations (page 18, lines 342–351; page 19, lines 352–382).

9. Figures and Tables: Figures summarizing caregiver and HCP satisfaction could benefit from clearer legends and consistent formatting of percentages.

Thank you for this suggestion. Upon further consideration, we removed the figure because the number of HCPs was small, and percentage-based visualization could be misleading. The findings are now presented descriptively in the Results, and Tables 2 and 3 now include all five Likert response categories, including zero-response categories (page 12, lines 226–230; page 13, lines 247–259).

10. Methodological Detail: Please provide more information on questionnaire validation and how qualitative response s were coded.

Thank you. We have expanded the Methods section to describe questionnaire pretesting and clarified that open-ended responses were not subjected to formal qualitative coding but were reviewed and descriptively summarized to identify commonly reported issues and experiences (page 8, lines 143–157; page 9, lines 158–168).

11. Terminology Consistency: Ensure consistent use of abbreviations (e.g., EIR, EPI) throughout the manuscript.

Thank you. We have reviewed the manuscript and standardized abbreviations, including EIR, EPI, RCC, LMICs, and HCPs, throughout the manuscript.

12. Ethical and Publication Considerations: Ethical approval and informed consent are clearly stated; no concerns identified.

Thank you. We also have strengthened the Ethics section by adding the ethics approval reference number and data confidentiality procedures (page 9, lines 170–175).

13. No evidence of dual publication or ethical violations.

Thank you for confirming this.

14. Financial disclosure and competing interests are appropriately declared.

Thank you.

15. Overall Recommendation: This manuscript makes a meaningful contribution to the literature on digital health and immunization in LMICs. With revisions to strengthen methodological transparency, expand discussion of limitations, and improve data accessibility, the paper will be well-positioned for publication.

Thank you for this encouraging recommendation. We have revised the manuscript to strengthen methodological transparency, expand limitations, avoid causal overinterpretation, improve reporting of Likert-scale data, and update the Data availability statement.

Reviewer #2

16. This manuscript presents a cross-sectional assessment of the feasibility and acceptability of an electronic immunization registry (EIR) in urban Bangladesh. The topic is relevant, the study appears technically sound, and the conclusions are generally supported by the data. However, several points require clarification.

Thank you for your comment. We have addressed each clarification point below.

17. Funding statement: There is an inconsistency between the Funding and Acknowledgements sections. The authors should clarify whether WHO-IVD support was financial or in-kind and ensure consistency between these sections.

Thank you for this comment. In response to the journal’s technical check following the revised submission, funding-related text has now been removed from the manuscript file, including the Funding, Competing Interests, and Acknowledgements sections. The Acknowledgements section in the revised manuscript now reads: “We thank the study participants for their contribution to this study.”

18. Data availability: For a survey-based study, PLOS data policy typically requires availability of the underlying de-identified dataset. The authors should clarify whether the raw survey data can be shared and update the Data Availability statement accordingly.

Thank you. We have updated the Data availability statement to indicate that the de-identified dataset is available as a supplementary file (page 21, lines 400–404)

19. The description of the Likert scale is inconsistent (described as 5-point but defined from 0 to 5) and should be corrected.

Thank you for identifying this error. We have now corrected the Likert-scale description throughout the manuscript. The scale is now consistently described as a 5-point scale ranging from 1 to 5 (page 8, lines 151–157; page 12, lines 227–230; page 14, lines 249–252).

20. Satisfaction outcomes are reported without explicit dissatisfied categories; the authors should clarify how these were handled and how overall satisfaction was calculated.

Thank you for this comment. We have revised Tables 2 and 3 to display all five Likert categories, including categories with zero responses. We also clarified that Likert items were analyzed individually and were not combined into a composite or overall satisfaction score (page 9, lines 159–168; page 12, lines 226–230; page 13–14, lines 247–252).

21. Minor typographical and formatting issues should be addressed.

Thank you. We have reviewed the manuscript and corrected typographical, grammatical, and formatting issues.

Reviewer #3

22. Study design and causal interpretation: The cross-sectional design of the study limits the ability to attribute the observed high vaccination coverage and improved registration timeliness directly to the electronic immunization registry. Several statements in the Abstract, Results, and Conclusions sections appear to imply an impact of the EIR rather than focusing strictly on feasibility and acceptability. The conclusions should be reframed to clearly distinguish association from causation and to avoid over-claiming system effectiveness.

Thank you. We agree and have revised the Abstract, Methods, Results, Discussion, and Conclusion to remove causal language and to frame the findings as descriptive perceptions and reported operational experiences rather than evidence of effectiveness or system-level impact (page 2–3, lines 31–58; page 7, lines 114–123; page 15–16, lines 277–295; page 20, lines 383–388).

23. Sampling strategy and potential bias: Caregivers were selected using purposive sampling, and all participants were already engaged with the EIR system. This approach introduces selection bias and likely inflates estimates of satisfaction and vaccination coverage. The limitations section should be expanded to explicitly discuss how purposive sampling and the exclusion of non-users may have influenced the findings.

Thank you. We have now clarified the purposive sampling strategy in the Methods and explicitly acknowledged selection bias in the limitations (page 8, lines 136–142; page 17, lines 317–320)

24. Vaccination coverage results (100% coverage): The reporting of 100% vaccination coverage for multiple antigens in an urban setting is unusual and requires careful contextualization. It is unclear whether the reported coverage reflects true population-level coverage or only coverage among children registered in the EIR system. The authors should clarify the denominators used, explicitly state that coverage estimates apply only to registered children, and temper interpretations accordingly.

We appreciate your concern and agree that the coverage results require careful contextualization. We have now clarified that these estimates reflect reported vaccination status among children registered in the EIR system and should not be interpreted as population-level vaccination coverage. We also have revised the Results and Discussion to avoid population-level interpretation (page 10, lines 183–190; page 11, lines 194–203).

25. Very small healthcare provider sample size: Only 16 healthcare providers were included in the study, yet results are presented in percentages and used to support relatively strong conclusions. This small sample size limits both statistical reliability and interpretive robustness. The manuscript should more explicitly acknowledge this limitation and avoid broad generalizations based on these data.

Thank you. We agree and now explicitly state that the HCP sample was small and that findings should be interpreted as indicative of provider perceptions within this setting. We have also removed broad claims based on HCP percentages (page 13, lines 231–242; page 17, lines 321–322)

26. Data availability statement: The manuscript states that all relevant data are contained within the manuscript, yet the results appear to be derived from underlying survey datasets. This may not fully align with PLOS ONE data transparency requirements. The authors should clarify whether anonymized datasets can be shared as supplementary files or deposited in a public repository.

Thank you. We have revised the Data availability statement to indicate that the de-identified dataset is available as a supplementary file (page 21, lines 400–404)

27. Likert scale description: The Likert scale is described in the Methods section as ranging from 0 to 5, while the tables appear to reflect a 1 to 5 scale. This discrepancy should be clarified and standardized throughout the manuscript.

Thank you for identifying this error. We have now corrected the Methods and table footnotes to describe the 5-point Likert scale consistently as 1 to 5 (page 8, lines 151–157; page 12, lines 227–230; page 14, lines 249–252).

28. Figures and tables: There are inconsistencies in figure numbering in the text, such as references to Figure 1 and Figure 3. These should be checked and corrected. In addition, figure captions should be improved to be fully self-explanatory without requiring reference to the main text.

Thank you for this suggestion. We have checked figure numbering and removed the figure from the manuscript, as it added limited analytical value and could be misleading given the small number of HCPs. The relevant findings are now presented descriptively in the Results section (page 14, lines 254–259).

Reviewer #4

29. Overall Assessment: This manuscript presents a cross-sectional evaluation of the feasibility and acceptability of an Electronic Immunization Registry (EIR) piloted in an urban setting in Bangladesh. The topic is timely and relevant, particularly in the context of digital health scale-up in low- and middle-income countries (LMICs) aligned with Immunization Agenda 2030. The study contributes descripti

Attachment Submitted filename: Response_to_Reviewers.docx

# Editor report: Decision Letter 1

**DOI:** 10.1371/journal.pone.0352972.r003

**Authors:** Samuel Kofi Tchum

18 Jun 2026

Feasibility and acceptability of an electronic immunization registry in urban Bangladesh

PONE-D-25-67616R1

Dear Dr. Uzzaman,

We’re pleased to inform you that your manuscript has been judged scientifically suitable for publication and will be formally accepted for publication once it meets all outstanding technical requirements.

Within one week, you’ll receive an e-mail detailing the required amendments. When these have been addressed, you’ll receive a formal acceptance letter and your manuscript will be scheduled for publication.

An invoice will be generated when your article is formally accepted. Please note, if your institution has a publishing partnership with PLOS and your article meets the relevant criteria, all or part of your publication costs will be covered. Please make sure your user information is up-to-date by logging into Editorial Manager at Editorial Manager® and clicking the ‘Update My Information' link at the top of the page. For questions related to billing, please contact billing support.

If your institution or institutions have a press office, please notify them about your upcoming paper to help maximize its impact. If they’ll be preparing press materials, please inform our press team as soon as possible -- no later than 48 hours after receiving the formal acceptance. Your manuscript will remain under strict press embargo until 2 pm Eastern Time on the date of publication. For more information, please contact onepress@plos.org.

Kind regards,

Samuel Kofi Tchum, Ph.D.

Academic Editor

PLOS One

Additional Editor Comments (optional):

Reviewers' comments:

# Editor report: Acceptance letter

**DOI:** 10.1371/journal.pone.0352972.r004

**Authors:** Samuel Kofi Tchum

PONE-D-25-67616R1

PLOS One

Dear Dr. Uzzaman,

I'm pleased to inform you that your manuscript has been deemed suitable for publication in PLOS One. Congratulations! Your manuscript is now being handed over to our production team.

At this stage, our production department will prepare your paper for publication. This includes ensuring the following:

* All references, tables, and figures are properly cited

* All relevant supporting information is included in the manuscript submission,

* There are no issues that prevent the paper from being properly typeset

You will receive further instructions from the production team, including instructions on how to review your proof when it is ready. Please keep in mind that we are working through a large volume of accepted articles, so please give us a few days to review your paper and let you know the next and final steps.

Lastly, if your institution or institutions have a press office, please let them know about your upcoming paper now to help maximize its impact. If they'll be preparing press materials, please inform our press team within the next 48 hours. Your manuscript will remain under strict press embargo until 2 pm Eastern Time on the date of publication. For more information, please contact onepress@plos.org.

You will receive an invoice from PLOS for your publication fee after your manuscript has reached the completed accept phase. If you receive an email requesting payment before acceptance or for any other service, this may be a phishing scheme. Learn how to identify phishing emails and protect your accounts at https://explore.plos.org/phishing.

If we can help with anything else, please email us at customercare@plos.org.

Thank you for submitting your work to PLOS ONE and supporting open access.

Kind regards,

PLOS ONE Editorial Office Staff

on behalf of

Dr Samuel Kofi Tchum

Academic Editor

PLOS One
